# A 33-residue peptide tag increases solubility and stability of *Escherichia coli* produced single-chain antibody fragments

**DOI:** 10.1038/s41467-022-32423-9

**Published:** 2022-08-08

**Authors:** Yang Wang, Wenjie Yuan, Siqi Guo, Qiqi Li, Xiaomei Chen, Cheng Li, Qianying Liu, Lei Sun, Zhenguo Chen, Zhenghong Yuan, Cheng Luo, Shijie Chen, Shuping Tong, Michael Nassal, Yu-Mei Wen, Yong-Xiang Wang

**Affiliations:** 1 Key Laboratory of Medical Molecular Virology (MOE/NHC/CAMS), School of Basic Medical Sciences, Shanghai Medical College, Fudan University, Shanghai, China; 2 Drug Discovery and Design Center, the Center for Chemical Biology, State Key Laboratory of Drug Research, Shanghai Institute of Materia Medica, Chinese Academy of Sciences, Shanghai, China; 3 School of Pharmacy, Nanchang University, Nanchang, China; 4 Institutes of Biomedical Science, Fudan University, Shanghai, China; 5 School of Pharmaceutical Science and Technology, Hangzhou Institute for Advanced Study, UCAS, Hangzhou, China; 6 Department of Internal Medicine II/Molecular Biology, University Hospital Freiburg, Freiburg, Germany

**Keywords:** Expression systems, Hepatitis B virus, Antibody fragment therapy, Chaperones, Proteins

## Abstract

Single-chain variable fragments (scFvs), composed of variable domains of heavy and light chains of an antibody joined by a linker, share antigen binding capacity with their parental antibody. Due to intrinsically low solubility and stability, only two *Escherichia coli*-produced scFvs have been approved for therapy. Here we report that a 33-residue peptide, termed P17 tag, increases the solubility of multiple scFvs produced in *Escherichia coli* SHuffle strain by up to 11.6 fold. Hydrophilic sequence, especially charged residues, but not the predicted α-helical secondary structure of P17 tag, contribute to the solubility enhancement. Notably, the P17 tag elevates the thermostability of scFv as efficiently as intra-domain disulfide bonds. Moreover, a P17-tagged scFv targeting hepatitis B virus surface proteins shows over two-fold higher antigen-binding affinity and virus-neutralizing activity than the untagged version. These data strongly suggest a type I intramolecular chaperone-like activity of the P17 tag. Hence, the P17 tag could benefit the research, production, and application of scFv.

## Abstract (web summary)

Low solubility and stability of *Escherichia coli* produced single chain variable fragments (scFvs) restrict their applications. Here the authors report a 33-residue peptide tag which simultaneously increases the solubility and thermostability of multiple scFvs produced in *Escherichia coli* SHuffle strain.

## Introduction

Antibody (Ab) is composed of two identical heterodimers of a heavy (H) chain and a light (L) chain. Each chain contains at least two types of immunoglobulin domains, i.e., one amino (N)-terminal variable domain and one or multiple carboxyl (C)-terminal constant domains. Due to high homogeneity and specificity, and strong affinity to antigens, monoclonal antibodies (mAbs) are widely used for disease diagnosis and therapy. Therapeutic applications of mAb rely heavily on pharmacokinetic properties, including rates of vesicular extravasation, tissue distribution, and clearance^1^. The large size (~150 kDa) of mAb restricts extravasation and distribution, reducing the effective mAb concentration within target tissues^1–3^. Due to long (~3 week) serum half-life^4^, prolonged retention of mAb conjugated with radioisotopes or toxins may lead to unexpected pathogenicity and toxicity. Besides, mAbs may extensively activate lymphocytes via interaction of their crystallizable fragments (Fc) with cell-surface receptors, resulting in adverse effects, e.g., cytokine storm^5^. These drawbacks are avoided by Ab fragments, such as antigen binding fragments (Fab) and variable fragments (Fv), which maintain the antigen specificity of their parental mAbs, but have Fc or the entire constant domains of both H and L chains deleted^6^. Due to their smaller sizes, Ab fragments have higher tissue penetration capability and are more rapidly cleared than mAbs^3,7^, making them particularly suitable for applications such as diagnostic in vivo imaging and therapeutic targeting of tumors and intracellular infectious agents, including viruses. Moreover, in contrast to costly mAb production in mammalian cells, Ab fragments can be principally be produced in prokaryotes such as *Escherichia coli* (*E. coli*) or *Brevibacillus choshinensis* at a much lower cost and in higher yield^8^. Despite these small-size-related benefits, Fc-less Ab fragments, especially Fv segments, are less stable than mAbs and show an increased tendency to unfold and aggregate under thermal stress. This is mainly attributed to the lack of inter- and intra-chain disulfide bonds and additional non-covalent interactions provided by the Fc domain^8,9^. Aggregates of Ab fragments can induce undesired immunogenicity and toxicity, resulting in compromised pharmacokinetic and therapeutic properties^2^. Strategies to improve the stability of Fv segments include reinforcing the interface between the variable domains of H and L chains (VH and VL) via covalent linkages in some formats, such as dsFv or scFv where two Fvs are covalently linked by a disulfide bond^10^ or a flexible peptide^11^, and sc-dsFv where two Fvs are linked by a disulfide bond plus a peptide^12^. Short (5–10 aa) peptide linkers to connect VH with VL promote intermolecular VH–VL interactions (called domain-swapping) by sterically disfavoring intramolecular VH–VL interactions, giving rise to dimers (also termed diabodies)^13^. Additional strategies have been developed to improve solubility and stability of bacterially expressed scFvs, including directed evolution, rational design, co-expression of chaperones, and fusion with solubility-enhancing tags^8^. However, despite these efforts, until 2020 only eight Ab fragments, two scFvs of them produced in *E.coli*, have been approved for clinical use by the US Food and Drug Administration^8^.

Intradomain disulfide bonding between two β-sheets of a VH or VL domain is often important to stabilize scFv. Mutations of the involved cysteines increase the propensity for aggregation^14,15^. The cytoplasmic compartment of common *E. coli* strains not only lacks components to catalyze the formation of disulfide bonds but instead possesses active glutathione reductase (*gor*) and thioredoxin reductase (*trxB*) pathways to reduce disulfide bonds^16^. Hence most cytoplasmically expressed scFvs are obtained as non-functional insoluble aggregates. While the periplasmic space, in contrast, provides an oxidative environment and contains thiol-disulfide oxidoreductases such as DsbA and DsbB^17^, recombinant expression yields are mostly very low. Recently, soluble expression of some scFvs has been achieved in an engineered *E. coli* strain (termed SHuffle) which features a more oxidative and disulfide-promoting cytoplasmic environment due to inactivation of the *gor* and *trxB* pathways and the constitutive expression of the disulfide-bond isomerase DsbC^18–21^. Also, other *E. coli* strains co-expressing a redox-active enzyme (e.g., Erv1p, DsbB or VKOR) plus a disulfide-bond isomerase like DsbC or PDI have become available^22–25^. However, some scFvs still exhibit low solubility in these engineered *E. coli* strains^25^ and the low stability of scFvs remains problematic^8^.

As reported, folding of select scFvs is predominantly mediated by intrinsically strong VH–VL interactions, independent of intradomain disulfide bond^9,26^. Their at least partly soluble cytoplasmic expression in *E. coli* is thus not unexpected. Fusion with some protein tags such as maltose-binding protein (MBP), glutathione-S-transferase (GST), N utilization substance A (NusA) and thioredoxin (Trx) can further improve the solubility^27–29^. However, for many scFvs with weak VH–VL interactions, this is unlikely the case^28^. In addition, it remains tricky and laborious to refold insoluble scFvs into active forms using detergents and additives, although fusion to MBP may improve expression and refolding yields^28^. However, it is often mandatory to remove bulky tags like MBP to preserve the scFv’s antigen-binding and pharmacokinetic properties^30^. In turn, tag removal often destabilizes the scFv and induces non-functional aggregate formation^29,30^.

The P17 protein from the tail of T7 phage can target phage particles yet also other cargos including small molecules, proteins, siRNA, DNA polyplexes, and liposomes to hepatocytes in vitro and in vivo^31,32^. This is predominantly mediated by the association of a 33-amino acid (aa) P17 protein segment (named P17 peptide or P17 tag hereafter) with cell-surface low density lipoprotein receptor-related protein (LRP)^33^. We previously generated an scFv of mAb G12^18,34^, which targets the surface protein of hepatitis B virus (HBV), a virus with pronounced liver tropism. To improve hepatocyte targeting and uptake of G12-scFv^18^, here we attach the P17 tag to its C-terminus. When expressed in the *E. coli* SHuffle strain, the G12-scFv-P17 fusion protein displays much higher solubility than the untagged G12-scFv. Furthermore, the P17 tag also increases the solubility of three other scFvs in SHuffle cells, as revealed by the semiquantitative solubility assay. The current study aims to identify the sequence and structure determinants within the P17 peptide that are responsible for its solubility and stability-enhancing impact on scFvs, and to clarify the underlying mechanisms.

## Results

### P17 tag enhanced solubility of four different scFvs in *E. coli* SHuffle T7 strain

**Fig. 1:**
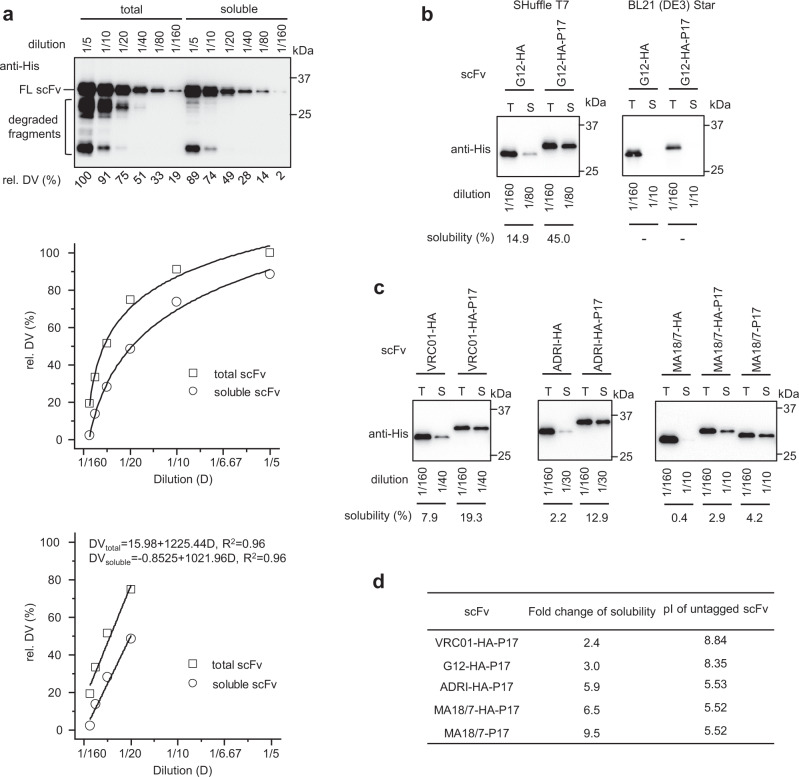
P17 tag enhanced solubility of four scFvs in *E. coli* SHuffle T7 cells. **a** Determination of dilution ranges of total and soluble fractions of G12-scFv-HA protein for linear correlation with western blotting signals. Upper panel: ScFvs from 5 μl total and soluble bacterial protein samples serially diluted with 2× SDS-PAGE loading buffer were detected by western blotting using an anti-His-tag. The relative density value (rel. DV) of each full-length (FL) scFv was obtained by setting the density of total scFv diluted by 1/5 at 100%. Middle panel: Over the entire dilution range rel. DV of both total and soluble scFvs correlated with dilutions logarithmically as calculated by the non-linear fitting program of Origin 8.0 software. Bottom panel: In the dilution range from 1/20 to 1/160, rel. DV of both total and soluble scFvs correlated linearly with dilutions as fulfilled by a linear fitting program of Origin 8.0. Linear equations of rel. DV and dilution (D) are also presented. **b** Determination of solubility of G12-scFv-HA and G12-scFv-HA-P17 proteins expressed in *E. coli* SHuffle T7 and BL21(DE3) Star strains. ScFv’s solubility was determined as the percentage ratio of soluble versus total scFvs after their dilutions were normalized at 1/160, and the result of one representative experiment is shown. − below the detection limit, T total protein, S soluble protein. **c** Determination of solubility of VRC01-scFv-HA, ADRI-scFv-HA, MA18/7-scFv-HA, and their P17-tagged derivatives expressed in SHuffle T7 strain. The result of one representative experiment was shown. **d** Fold change of solubility of P17-tagged scFvs to corresponding untagged scFvs and the isoelectric point (pI) of each untagged scFv.

Plasmid pET28a-His-G12-scFv-HA expresses a G12-scFv protein with a His tag on the N terminus and a HA tag at the C-terminus^18^. Into this plasmid, the P17 tag coding sequence was inserted downstream of the HA tag sequence, with the resulting plasmid pET28a-His-G12-scFv-HA-P17 encoding the same protein but now with the additional P17 tag at the C-terminus. *E. coli* SHuffle T7 cells transformed with either of the two plasmids were cultured in Luria-Bertani (LB) media supplemented with 0.5 mM IPTG to express recombinant G12-scFv-HA and G12-scFv-HA-P17 proteins, and subsequently were suspended in 10 ml lysis buffer per gram (wet weight) of bacteria. After cell rupture by sonification, 10 μl of each lysate containing total bacterial protein was mixed with 40 μl 2× SDS-PAGE loading buffer as the source of total scFv. After centrifugation, 10 μl of the clear lysate supernatant containing soluble bacterial protein was mixed with 40 μl 2× SDS-PAGE loading buffer as the source of soluble scFv. To determine the solubility of scFv defined as the percentage ratio of soluble to total scFv, both fractions of scFvs were semi-quantitated by western blotting using a mAb against the common His tag followed by a horseradish peroxidase-conjugated secondary antibody plus a chemiluminescent substrate, and subsequent densitometry of the blotting signals recorded by a charge-coupled device camera (Fig. 1a, upper panel). To avoid signal saturation, both fractions of scFvs were diluted serially by 1/5, 1/10, 1/20, 1/40, 1/80, and 1/160 before loading. As shown in Fig. 1a (middle panel), western blotting signals of both fractions of scFvs correlated logarithmically with the dilution. However, at high dilutions ranging from 1/160 to 1/20, signal intensities displayed an approximately linear correlation with the dilution (Fig. 1a, bottom panel). Therefore, for quantitation, soluble scFv was diluted in the range from 1/20 to 1/80, while total scFv was diluted by 1/160 due to the higher amount. ScFv solubility was assessed after normalizing the dilution of soluble scFv at 1/160. As shown in the left panel of Fig. 1b, the solubility of G12-scFv-HA and G12-scFv-HA-P17 proteins was 14.9% and 45.0%, respectively, indicating the P17 tag increased G12-scFv solubility.

In line with our previous report^18^, the G12-scFv-HA protein was virtually insoluble when expressed in BL21 (DE3) Star cells, which harbor active *gor* and *trxB* pathways preventing intradomain disulfide bond formation (Fig. 1b, right panel). Also, the G12-scFv-HA-P17 protein remained insoluble in this strain (Fig. 1b, right panel), indicating that the solubility-enhancing effect of the P17 tag required an oxidative cytoplasmic environment.

To check whether the P17 tag could enhance the solubility of other scFvs, recombinant pET28-derived plasmids were constructed to express three additional scFvs: VRC01-scFv targeting gp120 of HIV-1^35^, ADRI-scFv targeting the small surface protein of HBV^36^, and MA18/7-scFv targeting preS1 domain of the large HBV surface protein^37^, either with or without the P17 tag at their C termini. As shown in Fig. 1c, d, the solubility of these scFvs expressed in SHuffle T7 strain showed some variations, but fusion with the P17 tag increased in all cases solubility from 2.4 to 6.5-fold. The solubility-promoting effect of the P17 tag was most remarkable for MA18/7-scFv, with its untagged version having the lowest solubility (Fig. 1d).

In the scFv constructs described above, a HA tag was located between scFv and P17 tag to facilitate immunological detection. To check whether the HA tag contributed to the solubility-enhancing effect of the P17 tag, we constructed an analogous plasmid, pET28a-His-MA18/7-scFv-P17, which lacks the HA coding sequence. In direct comparison with MA18/7-scFv-HA, the solubility of this new construct, MA18/7-scFv-P17, was even more elevated (by 9.5-fold) (Fig. 1c, d), indicating that the additional presence of the HA tag decreased solubility enhancement by the P17 tag.

### Both N- and C-terminal fusions of the P17 tag efficiently increased scFv solubility

**Fig. 2:**
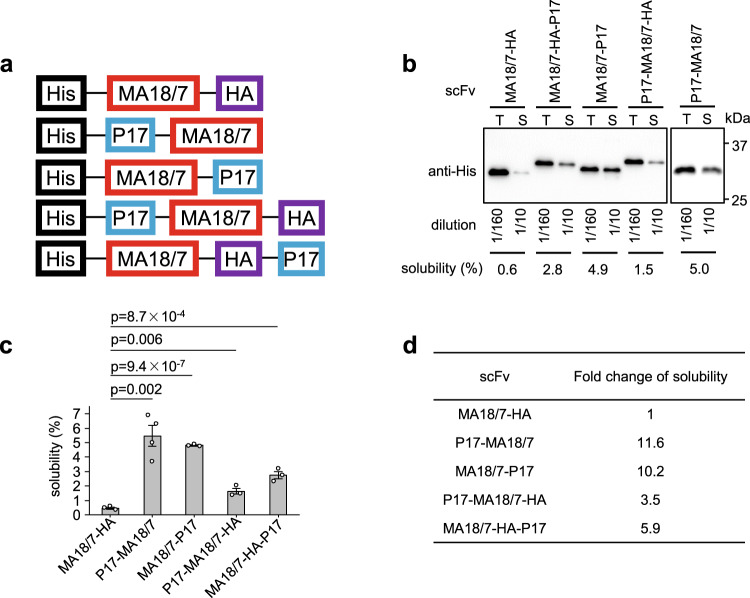
P17 tag significantly improved solubility of scFv in SHuffle T7 strain regardless of fusion positions. **a** Schematic diagrams of His-MA18/7-scFv-HA protein and its N- and C-terminally P17-tagged derivatives with a C-terminal HA tag fusion or not. **b** Determination of solubility of His-MA18/7-scFv-HA protein and its derivatives expressed in SHuffle T7 cells by western blotting. The result of one representative experiment was shown. T, total protein; S, soluble protein. **c** Solubility of His-MA18/7-scFv-HA and its derivatives were determined by three independent experiments. Data are presented as mean values ± SD. The statistical significance of differences between two experimental groups was assessed through one-way analysis of variance (ANOVA). **d** Fold change of solubility of P17-tagged derivatives of His-MA18/7-scFv-HA to untagged version.

The impact of a tag on the solubility of a fusion protein can be affected by the tag’s location at the N or C-terminus of a protein of interest^38^. Therefore, we compared the solubility of MA18/7-scFv with the P17 tag directly linked to either end (Fig. 2a). As shown in Fig. 2b–d, the P17 tag significantly improved solubility at both N- and C-positions by 11.6- and 10.2-fold, respectively. In the additional presence of a HA tag at the C-terminus, the P17 tag increased solubility by 3.5-fold when at the N terminus and 5.9-fold when at the very C-terminus (Fig. 2d). Therefore, direct attachment of the P17 tag to either terminus of the scFv increased solubility efficiently, whereas an additional HA tag at the scFv C-terminus reduced the P17-mediated solubility enhancement.

### A C-terminal P17 tag enhanced G12-scFv solubility more efficiently than N- or C-terminally fused MBP in SHuffle T7 cells

**Fig. 3:**
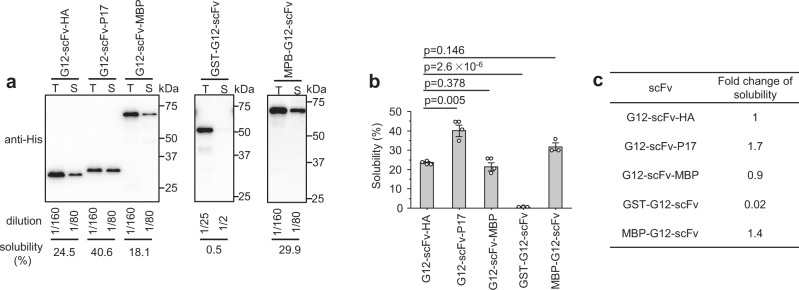
A C-terminal P17 tag increased scFv solubility in SHuffle T7 cells more efficiently than N- or C-terminal MBP and N-terminal GST. **a** Determination of solubility of each G12-scFv fusion protein with HA and P17 tag attached at C-terminus, MBP tag at N or C-terminus, or GST tag at N terminus by western blotting. T, total protein; S, soluble protein. **b** Solubility of G12-scFv fusion proteins was determined by three independent experiments. **c** Fold change of solubility of P17, MBP, and GST-tagged scFvs compared to HA-tagged scFv. Data are presented as mean values ± SD. The statistical significance of differences between the two experimental groups was assessed by one-way ANOVA.

Multiple fusion tags, including MBP, have been applied to improve the solubility of scFvs in *E. coli*^27–29^. Indeed, N-terminal but not C-terminal MBP fusion increased the solubility of G12-scFv by 1.4-fold in SHuffle T7 cells (Fig. 3c) as revealed by the solubility assay (Fig. 3a, b). C-terminal P17 tag fusion increased the solubility of G12-scFv by 1.7-fold, in line with a higher soluble yield of G12-scFv-P17 than G12-scFv-HA and MBP-G12-scFv (Supplementary Fig. S3b). Hence in SHuffle T7 cells, the P17 tag increased the solubility of G12-scFv more efficiently than MBP. Glutathione-S-transferase (GST) is another protein tag that is commonly used to improve the solubility of passenger proteins in *E. coli*^39^, often as N-terminal fusion^40^. Its effect on the solubility of G12-scFv was therefore also tested. However, the GST-G12-scFv-HA protein was even much less soluble in SHuffle T7 cells than the N-terminally untagged version (Fig. 3a–c).

### Adding two or three copies of P17 tags did not further improve scFv solubility

**Fig. 4:**
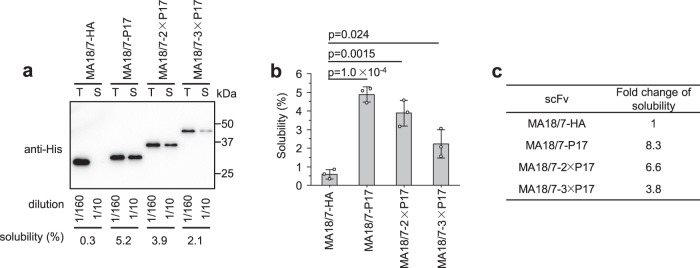
Effects of fusion of one, two, or three C-terminal P17 tag(s) on scFv’s solubility in SHuffle T7 cells. **a** Determination of solubility of MA18/7-scFv-HA protein and MA18/7-scFv fusion protein with one, two, and three P17 tag(s) individually attached at the C-terminus by western blotting. T total protein, S soluble protein. **b** Solubility of MA18/7-scFv-HA and three other MA18/7-scFv fusion proteins were determined by three independent experiments. **c** Fold change of solubility of scFvs fused to different numbers of P17 tags to untagged one. Data are presented as mean values ± SD. The statistical significance of differences between the two experimental groups was assessed by one-way ANOVA.

The solubility-enhancing ability of the P17 tag may be endowed by recruiting other molecules such as chaperones or perhaps tRNAs^41–44^ to promote protein folding. If so, adding two or three copies of the P17 tag might further elevate the solubility of a test scFv. To test this hypothesis, two or three copies of the tag were fused to the C-terminus of MA18/7-scFv. The results showed that a single P17 tag increased solubility by 8.3-fold, whereas two and three copies enhanced solubility by only 6.6- and 3.8-fold, respectively (Fig. 4).

### A predicted α-helix in the P17 tag is not required for solubility enhancement

**Fig. 5:**
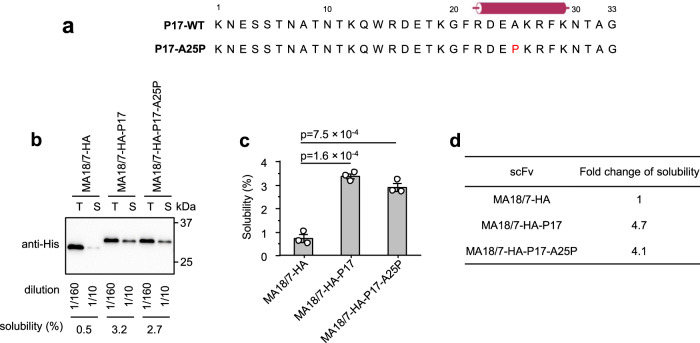
A putative α-helix in the P17 tag was not required for solubility enhancement. **a** Amino acid sequences of wild-type (wt) P17 tag (P17-WT) and it's mutant (P17-A25P) with alanine 25 substituted by proline. The predicted α-helix formed by P17 tag residues 22–29 is depicted as a cylinder. Proline 25 of P17-A25P mutant is shown in red. **b** Representative western blot to determine the solubility of MA18/7-scFv-HA protein and its two derivatives with C-terminally fused P17-WT and P17-A25P tags. T total protein, S soluble protein. **c** Solubility of MA18/7-scFv-HA protein and its derivatives determined by three independent experiments. **d** Fold change of solubility of MA18/7-scFv-HA-P17 and MA18/7-scFv-HA-P17-A25P proteins compared to MA18/7-scFv-HA protein. Data are presented as mean values ± SD. The statistical significance of differences between the two experimental groups was assessed by one-way ANOVA.

**Fig. 6:**
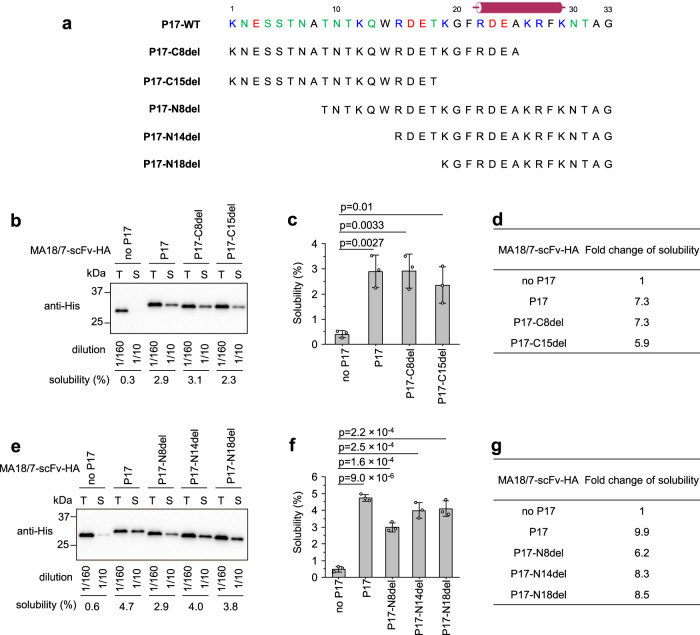
Both N and C-terminal sequences of the P17 tag contribute to enhanced scFv solubility in SHuffle T7 cells. **a** Amino acid sequences of wt P17 tag (P17-WT) and its truncated mutants with various deletions of C- or N-terminal residues. Basic, acidic, and polar residues are shown in blue, red, and green, respectively. The putative α-helix from P17 residues 22–29 is denoted with a cylinder. **b** Representative western blot to determine the solubility of MA18/7-scFv-HA protein and its derivatives fused to P17-WT or the C-terminally truncated mutants P17-C8del and P17-C15del. **c** Semiquantitative determination of the solubility of MA18/7-scFv-HA protein and its derivatives from **b** by three independent experiments. **d** Fold change of solubility of MA18/7-scFv derivatives from b compared to untagged MA18/7-scFv-HA. **e** Representative western blot to determine the solubility of MA18/7-scFv-HA protein and its derivatives fused to P17-WT or the N-terminally truncated mutants P17-N8del, P17-N14del, and P17-N18del. **f** Semiquantitative determination of the solubility of MA18/7-scFv-HA protein and its derivatives from **e** by three independent experiments. **g** Fold change of solubility of MA18/7-scFv derivatives from e compared to untagged MA18/7-scFv-HA. T total protein, S soluble protein. Data are presented as mean values ± SD. The statistical significance of differences between the two experimental groups was assessed by one-way ANOVA.

Structural features of a protein tag can be important for solubility enhancement^45,46^. The free P17 peptide did not exhibit any characteristic α-helical structure when analyzed by circular dichroism spectroscopy^31^, but an earlier study of the entire P17 protein had noted a substantial α-helix propensity in the rod-region harboring the P17 tag sequence^47^. In the absence of direct structural information, we applied up-to-date secondary structure prediction algorithms, JPred4^48^ and PEP2D^49^, to the P17 peptide (see Supplementary Fig. S1a for the JPred4 results), and the three-dimensional (3D) structure prediction program AlphaFold2^50^ to the entire MA18/7-scFv-HA-P17 protein (Supplementary Fig. S1b). Either program predicted a single region within the P17 tag to have some α-helix propensity, namely residues 22–29 (RDEAKRFK, Fig. 5a) near the C-terminus, although the AlphaFold2 predicted local Distance Difference Test (plDDT) score in this region was below 70 (Supplementary Fig. S1b), indicating an at most moderate confidence in the predicted α-helix. However, the α-helix might still form in the scFv context or be induced by interaction with bacterial components, notably the abundant tRNA, as in the recently described “chaperna” mechanism where RNA is crucial for chaperoning^41–44^. Different from the RNA interaction domain used there, we could not detect a stable association of the P17 tag with RNA (Supplementary Fig. S2), disfavoring such a mechanism. To further experimentally assess whether the putative α-helix, if formed, was important for solubility enhancement by the P17 tag, we replaced the alanine in the center of the putative helix, A25, by a helix-breaking proline residue^51^ (Fig. 5a, variant A25P); JPred4, as well as alphafold2, predicted indeed a loss of the α-helix (Supplementary Fig. S1a, b, variant A25P). When attached to the C-terminus of MA18/7-scFv-HA, both the wild-type (wt) and A25P mutant P17 tag increased protein solubility by around fourfold (Fig. 5b–d). Furthermore, a variant lacking the entire sequence forming the putative α-helix (variant P17-C15del), was at most slightly less efficient than the wt P17 tag to increase the solubility of MA18/7-scFv (see below, Fig. 6a–d). Therefore, the putative α-helix structure, if formed at all, was dispensable for solubility enhancement.

### Both N- and C-proximal sequences in the P17 tag contribute to scFv solubility enhancement

The 33-aa P17 tag is highly hydrophilic due to the presence of eight basic aa (R, K), five acidic aa (D, E), and twelve polar aa (e.g., N, Q, S, T), all of which may contribute to the tag’s solubility-enhancing property. As these hydrophilic residues are scattered throughout the sequence (Fig. 6a), we truncated the P17 tag from either terminus (Fig. 6a) to define the relevant sequence element(s).

P17 mutants P17-C8del and P17-C15del lack 8 and 15 aa from the C-terminus to partly and completely delete the sequence forming the predicted α-helix. Attached to the C-terminus of MA18/7-scFv both improved solubility significantly by 7.3- and 5.9-fold, respectively (Fig. 6b–d), only slightly less than the wt P17 tag (Fig. 6d). P17 mutants P17-N8del, P17-N14del, and P17-N18del lacking 8, 14, and 18 aa from the N terminus also increased solubility of MA18/7-scFv significantly by 6.2-, 8.3-, and 8.5-fold, respectively (Fig. 6e–g). Notably, P17-N18del maintains only 15 aa of the P17 tag but promoted scFv solubility as strongly as the wt tag (Fig. 6g). Taken together, both N- and C-terminal sequences of the P17 tag can independently improve scFv’s solubility in the SHuffle T7 strain.

### The solubility-enhancing ability of the P17 tag correlated with the number of charged residues

**Fig. 7:**
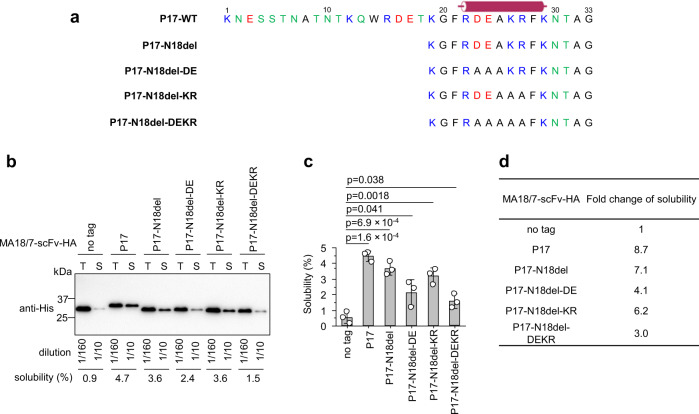
Alanine substitution of basic and acidic residues reduced solubility-enhancing ability of the P17-N18del tag. **a** Amino acid sequences of wt P17 tag (P17-WT), truncated mutant P17-N18del and its point mutants P17-N18del-DE, P17-N18del-KR, and P17-N18del-DEKR. In these mutants, acidic residues (D23 and E24) and basic residues (K26 and R27) were replaced alone or in combination by alanine (A). Basic, acidic, and polar residues are shown in blue, red, and green, respectively. Residues 22– 29 of P17 tag predicted to form α-helix are denoted with a cylinder. **b** Representative western blot to determine the solubility of MA18/7-scFv-HA protein and its derivatives fused to P17-WT, P17-N18del, or the indicated point mutants of P17-N18del. T total protein, S soluble protein. **c** Semiquantitative determination of the solubility of MA18/7-scFv-HA protein and its derivatives from by three independent experiments. Data are presented as mean values ± SD. The statistical significance of differences between the two experimental groups was assessed by one-way ANOVA. **d** Fold change of solubility of MA18/7-scFv derivatives from a compared to untagged MA18/7-scFv protein.

Polyanionic and polycationic protein tags have been reported to improve the solubility of some recombinant proteins^46^. As 15-aa P17-N18del retained wt-like solubility-enhancing ability, it was used for subsequent point-mutation analysis. Because two anionic residues (D23, E24) and two cationic residues (K26, R27) are clustered in the central region of P17-N18del (Fig. 7a), we investigated their potential roles by site-directed mutants (P17-N18del-KR, P17-N18del-DE, and P17-N18del-DEKR) with D23/E24 and/or K26/R27 replaced alone or in combination by alanine (Fig. 7a) were fused to the C-terminus of MA18/7-scFv. In the context of the P17 tag, all mutants were predicted to maintain a wt-like α-helix propensity between residues R22-K29 (Supplementary Fig. S1a, there named P17 DE23,24AA and KR26,27AA). Hence, these variants would also test for the importance of positive and negative charges in the putative α-helix, even if its formation was unlikely (see above). All these point mutants still increased solubility of MA18/7-scFv significantly, though to variable extents (Fig. 7b–d). Compared to the parental P17-N18del tag, the solubility-enhancing abilities of P17-N18del-KR, P17-N18del-DE, and P17-N18del-DEKR mutants were reduced by 12%, 42%, and 58%, respectively (Fig. 7d). Therefore, the clustered charged residues have the strongest impact on the solubility-enhancing ability of the P17 tag.

### The P17 tag increases scFv stability

**Fig. 8:**
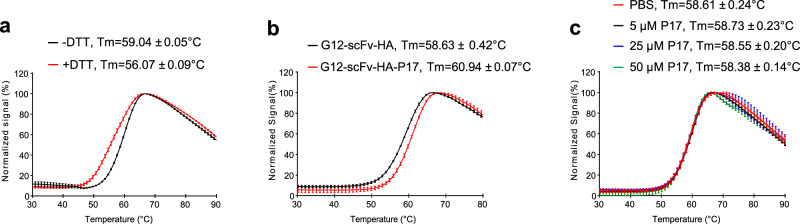
P17 tag increased thermostability of G12-scFv. **a** Reduction of intradomain disulfide bonds of G12-scFv-HA protein by DTT decreased melting temperature (Tm). Five μM G12-scFv-HA protein, treated with 5 mM DTT for 30 min at room temperature or not, was mixed with 1× SYPRO Orange. The fluorescence signal emitted at 570 nm by SYPRO Orange at each temperature was recorded by a QuantStudio PCR apparatus and normalized to the maximal signal reached which was set at 100%. Tm of scFv was determined by three independent thermal shift assays as described in “Methods”. **b** Tm of G12-scFv-HA and G12-scFv-HA-P17 proteins. **c** Tm of G12-scFv-HA protein at the presence of in vitro synthesized P17 peptide. After separately mixing with 5 μM, 25 μM, and 50 μM P17 peptide, Tm of 5 μM of G12-scFv-HA protein was measured three times by thermal shift assay. Results shown by average fluorescence values and error bars at each temperature were plotted using Origin 8.0 software.

Protein tags can enhance solubility by assisting in the correct folding of the fusion partners^45,46^. This reduces exposure of hydrophobic regions to solvent and inhibit formation of aggregates. Protein stability decreases with increasing temperature and can be reflected by the melting temperature (Tm) at which the concentrations of folded and unfolded proteins are equal^52^. Generally, a higher Tm indicates higher stability and vice versa^52^. As reported previously, the Tm of a test protein can be determined by a thermal shift assay^52,53^. In this assay, fluorescent dye (SYPRO Orange) binds to hydrophobic regions exposed during heat denaturation, yielding more 570-nm-wavelength quanta^53^. As determined by this assay, treatment of G12-scFv-HA with 5 mM DTT to reduce disulfide bonds led to ~3 °C decrease of Tm (Fig. 8a), in line with the presence of such disulfides and their importance of intradomain disulfide bonds to maintain scFv stability in previous studies^14,15^.

Next, to check the effect of P17 tag on scFv stability, recombinant G12-scFv-HA and G12-scFv-HA-P17 proteins were firstly purified by IMAC (Supplementary Fig. S3a). Quantitation of both proteins by SDS-PAGE and Coomassie-Blue staining using BSA as standard revealed soluble yields of 3 mg and 8 mg per liter culture (Supplementary Fig. S3b), corroborating the solubility enhancement by the P17 tag. Five μM solutions of both proteins were mixed with equal concentrations of SYPRO Orange and subjected to the thermal shift assay. As shown in Fig. 8b, G12-scFv-HA-P17 displayed a 2.3 °C higher Tm than G12-scFv-HA, indicating that the P17 tag stabilized scFv by inhibiting exposure of hydrophobic regions. As a control, incubation in trans with in vitro synthesized P17 peptide failed to increase the Tm of G12-scFv-HA even in 10-fold excess (Fig. 8c), indicating that the P17 tag stabilized scFv only as part of the protein and did so by increasing resistance against unfolding.

### P17-tagged G12-scFv has higher antigen binding affinity and specificity and HBV infection-neutralizing activity than the untagged protein

**Fig. 9:**
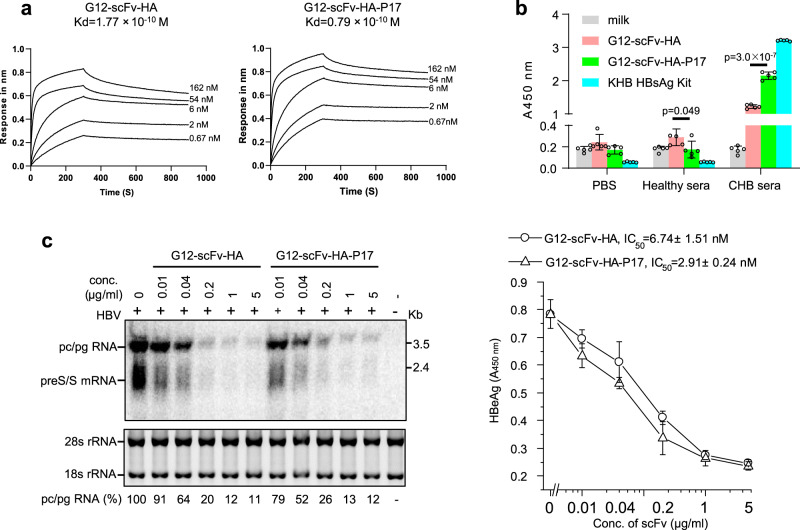
P17-tagged G12-scFv showed stronger antigen binding affinity and specificity and HBV-neutralizing activity than G12-scFv. **a** Biolayer interferometry to determine the binding affinity of G12-scFv-HA and G12-scFv-HA-P17 proteins to recombinant HBV small surface protein from CHO cells. Kd, equilibrium dissociation constant. **b** Enzyme-linked immunosorbent assay (ELISA) to test binding affinity and specificity of G12-scFv-HA and G12-scFv-HA-P17 proteins. The pooled sera of chronic hepatitis B patients and healthy donors were confirmed to be positive and negative for hepatitis B surface antigen (HBsAg) by a commercial KHB ELISA kit, and then reacted with the microtiter wells coated with 5 μg/ml solutions of G12-scFv-HA and G12-scFv-HA-P17, respectively. Data are presented as mean values ± SD. The statistical significance of differences between two experimental groups was assessed by one-way ANOVA. **c** Determination of HBV-neutralizing activity of G12-scFv-HA and G12-scFv-HA-P17 proteins by northern blotting and ELISA. HepG2-NTCP cells were inoculated overnight with 100 vge/cell HBV particles pre-incubated for 30 min with serially diluted G12-scFv-HA or G12-scFv-HA-P17 protein. After extensive washing, cells were cultured in DMEM containing 2.5% DMSO. Intracellular viral RNAs (left panel) and secreted HBeAg (right panel) in supernatants were detected at day 5 post-inoculation by northern blotting and ELISA, respectively. Pc RNA, precore mRNA; pgRNA, pregenomic RNA. For comparison, the signal intensity of pc/pgRNA was normalized to that of 28 s and 18 s rRNA (as loading controls). A_450 nm_ values for HBeAg subtracting the ELISA cutoff of 0.11 at various concentrations of scFvs, determined by four independent experiments, are shown. IC_50_ was calculated with Origin 8.0 software. Pc/pgRNA and HBeAg from untreated cells were set at 100%. +, with HBV inoculation; −, without HBV inoculation.

Due to its increased stability, the scFv-P17 fusion protein may possess stronger antigen-binding affinity than unfused scFv, unless the tag would sterically hinder antigen binding. To compare the binding affinity of G12-scFv-HA and G12-scFv-HA-P17 to recombinant HBV small surface protein produced in CHO cells, the equilibrium dissociation constants (Kd) were determined by biolayer interferometry. A 2.2-fold lower Kd (0.79 × 10^−10 ^M versus 1.77 × 10^−10 ^M, Fig. 9a) for G12-scFv-HA-P17 than for G12-scFv-HA was obtained. These results were further corroborated by enzyme-linked immunosorbent assay (ELISA), whereby the pooled sera of chronic hepatitis B patients and healthy donors, confirmed to be positive and negative for hepatitis B surface antigen (HBsAg) by a commercial ELISA kit (Fig. 9b), were reacted with the microtiter wells coated with 5 μg/ml solutions of G12-scFv-HA and G12-scFv-HA-P17, respectively. ELISA A450nm value of G12-scFv-HA-P17 reacting with HBsAg positive sera was significantly (*P* < 0.0001), approximately twofold higher than G12-scFv-HA (Fig. 9b). These data collectively indicate stronger antigen binding affinity of G12-scFv-HA-P17 than G12-scFv-HA, and also suggest that the P17 tag did not interfere with scFv-antigen binding. Generally, stronger antigen binding affinity of scFv suggests higher specificity. Indeed, ELISA A450nm value of G12-scFv-HA-P17 incubating with HBsAg negative sera was significantly (*P* < 0.05) lower than G12-scFv-HA, thus indicating the higher specificity (Fig. 9b). Importantly, both G12-scFv-HA and G12-scFv-HA-P17 proteins efficiently inhibited HBV infection of a susceptible cell line as judged by the dose-dependent suppression of HBV RNAs and secreted hepatitis B e antigen (Fig. 9c). However, G12-scFv-HA-P17 protein displayed higher neutralizing activity than G12-scFv-HA, as indicated by of 50% inhibitory concentrations at 2.91 nM versus 6.74 nM (Fig. 9c, right panel).

### P17 tag did not lead to oligomerization of G12-scFv

**Fig. 10:**
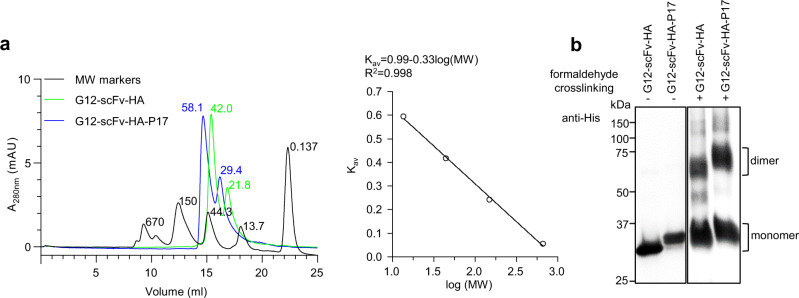
P17 tag did not induce oligomerization of G12-scFv. **a** Size-exclusion chromatography profiles of protein markers, G12-scFv-HA, and G12-scFv-HA-P17 proteins. Two hundred μl protein markers, G12-scFv-HA, and G12-scFv-HA-P17 proteins were loaded onto a Superdex 200 Increase 10/300 column using an ÄKTA purifier system. Proteins eluting by PBS at a flow rate of 0.5 ml/min were monitored by ultraviolet absorbance at 280 nm wavelength (A_280nm_). Elution peaks of protein markers (in black), including bovine thyroglobulin, bovine γ-globulin, chicken egg albumin, ribonuclease A, and p-aminobenzoic acid were labeled with corresponding molecular weights (MWs, 670 kDa, 150 kDa, 44.3 kDa, 13.7 kDa, and 0.137 kDa) in the left panel. Elution peaks of G12-scFv-HA (in green) and G12-scFv-HA-P17 (in blue) proteins were labeled with the MWs calculated from the calibration curve and equation in the right panel. Right panel: calibration curve and equation of average distribution constants (K_av_) of protein markers and logarithms of their MWs (log (MW)). **b** Western blotting results of G12-scFv-HA and G12-scFv-HA-P17 proteins treated with formaldehyde or not. Five hundred ng G12-scFv-HA and G12-scFv-HA-P17 proteins were treated with 3% formaldehyde at 37 °C for 10 min or not, followed by the addition of 5×SDS sample loading buffer, and were detected by western blotting using anti-His mAb following separation by SDS-10% PAGE. The result of one representative experiment was shown. −, without formaldehyde cross-linking; +, with formaldehyde cross-linking.

Some protein tags like GST mediate oligomerization of fusion partners by spontaneous self-assembly into dimer or higher oligomers^45,54^. Recombinant full-length P17 protein assembles into trimer, as natively present in T7 phage tail fibers^47^. In contrast, the 33-aa P17 peptide existed as a monomer in solution as determined by circular dichroism and analytical ultracentrifugation^31^. To assess the potential impact of the P17 tag on G12-scFv quaternary structure, recombinant G12-scFv-HA and G12-scFv-HA-P17 proteins were analyzed by size-exclusion chromatography (SEC). As shown in Fig. 10a (left panel), the Superdex 200 Increase 10/300 column separated protein markers ranging from 0.137 kDa to 670 kDa very well, giving the linear calibration curve and equation of average distribution constants (K_av_) of each protein marker and the logarithm of the respective molecular weight (Log(MW), Fig. 10a, right panel). Under identical experimental conditions, both the G12-scFv-HA and the G12-scFv-HA-P17 protein displayed two peaks (Fig. 10a, left panel), representing monomeric and dimeric forms of each protein based on the calibration curve and equation. To confirm this interpretation, both protein preparations were cross-linked using 3% (v/v) formaldehyde, and the products were analyzed by SDS-PAGE and subsequent western blotting using the anti-His-tag mAb (Fig. 10b). This revealed dimers as the major higher-order products. The ratios of dimer to monomer were essentially the same for G12-scFv-HA and G12-scFv-HA-P17, both by the intensity of the western blot signals and the areas of the SEC elution peaks (Fig. 10a, b). Taken together, the P17 tag neither resulted in oligomerization of G12-scFv nor did it alter the equilibrium between monomeric and dimeric forms, rather in line with a type I than an assembly-mediating type II intramolecular chaperone mechanism^55^.

## Discussion

As one format of antibody fragments, scFv are applied in the fields of basic research, diagnostics, and therapeutics. Amongst different expression hosts^56,57^, *E. coli* offers rapid growth at low cost^8^. However, scFv are often expressed as insoluble aggregates because common *E. coli* strains with their reducing cytoplasmic environment fail to support formation of intramolecular disulfide bond^16^. Strategies to improve scFv solubility in *E. coli* include expression in the oxidative periplasmic space, reduced culture temperature, co-expression of chaperone(s), using engineered *E. coli* variants providing a more oxidizing cytoplasm, and fusion of solubility-promoting tags to the scFv^2,8^. Here, we characterized the 33-aa P17 peptide tag, and further shortened variants, as a small-size fusion tag which markedly increases the solubility, soluble yield, and stability of scFvs in *E. coli* SHuffle cells.

### Solubility-enhancing effect of P17 tag on multiple scFvs

Analogous to other protein tags, solubility enhancement of scFvs by the P17 tag depends on both the expression host and properties of the specific scFv^45,58^. Firstly, in line with intradomain disulfide-bond formation promoting scFv solubility in *E. coli*, G12-scFv was partly soluble in the SHuffle strain but not in the BL21(DE3) strain with its reducing cytoplasm (Fig. 1b). Only in SHuffle cells did the P17 tag further increase scFv solubility (Fig. 1b, d). Secondly, while the P17 tag did not improve solubility of a truncated duck hepatitis B virus polymerase, notorious for poor solubility in *E. coli*^59^ (Supplementary Fig. S4), the tag enhanced solubility of four different scFvs (Fig. 1d). Part of Ab diversity is due to many subgroups of heavy and light chains. Heavy chains are encoded by a single gene cluster (IGH), light chains by the κ cluster (IGK) and the λ cluster (IGL)^60^. Sequence analysis of the four scFvs using online International ImMunoGeneTics Information System (http://www.imgt.org/) revealed that their variable regions are encoded by different subgroups of heavy (IGHV) and light chain variable gene clusters (IGKV, and IGLV). Accordingly, the variable regions of MA18/7-scFv are encoded by IGHV3 and IGKV6; those of G12-scFv by IGHV4 and IGKV3; those of VRC01-scFv by IGHV1 and IGKV1; and those of ADRI-scFv by IGHV3 and IGLV3. Although these scFvs vary considerably in sequence and their intrinsic solubility properties in *E. coli*, attaching the P17 tag improved the solubility of all by at least 2.4-fold and up to 11.6-fold (Figs. 1 and 2). Hence, the P17 tag can improve the solubility of multiple scFvs in the SHuffle strain.

### Properties of the P17 tag and implications

Their small sizes of around 30 kDa convey to scFvs efficient tissue penetration and renal glomerular filtration^3^. Oligomerization and the corresponding increase in size would substantially dampen these properties. However, the P17 peptide as such exists as monomer in solution and also as a tag it neither induced oligomerization of scFv nor did it affect the intrinsic scFv monomer-dimer equilibrium (Fig. 10). With about 4 kDa in size the P17 tag is much smaller than most other currently used protein tags. Bulkier tags impair size-related pharmacokinetic scFv properties and also increase the metabolic burden of the expression hosts. Furthermore, bulky tags such as MBP may sterically interfere with the scFv-antigen interaction^61^ since MBP-G12-scFv fusion protein displayed significantly lower binding affinity to recombinant HBV small surface protein than untagged G12-scFv-HA (Supplementary Fig. S5). Hence larger tags often need to be proteolytically removed^45^, which can destabilize scFvs^29,30^. In our study, the 33-aa P17 tag and its truncated variants, especially P17-N18del mutant comprising only 15 aa (Fig. 6g), improved scFv solubility regardless of terminal fusion positions (Fig. 6). The P17 tag displayed stronger solubility-enhancing effects on scFv than C-terminally fused MBP or N-terminally fused GST (Fig. 3). Most remarkably, the P17 tag also improved thermostability, antigen binding affinity and specificity of scFv (Figs. 8 and  9). Therefore, the P17 tag removal is not mandatory for in vitro use. However, if desired, especially for avoiding immunogenicity in in vivo applications, the P17 tag can be efficiently cleaved off by enterokinase, and possibly other cleavage site/protease combinations (Supplementary Fig. S3c) after the introduction of an enterokinase digestion site (DDDDK). Importantly, solubility of the tag-less scFv protein was not compromised, as shown by virtually identical band intensities of equal amounts of untreated vs. enterokinase-treated protein after centrifugation at 20,000×*g* for 30 min (Supplementary Fig. S3c). These data also suggest that the P17 tag facilitates the adoption of a stably folded scFv conformation but is not required to maintain it.

For therapeutic and diagnostic purposes, scFvs and diabodies have to maintain antigen-binding activity without aggregating at 37 °C for hours to days after injection, requiring high intrinsic stability^8,9^. The P17 tag not only promoted expression solubility in *E. coli* SHuffle strain but also increased thermostability and antigen binding affinity of G12-scFv. In this way, the P17 tag could benefit long-term storage stability of scFvs, as practically supported by the much fewer insoluble aggregates observed after 1.5 years of cryo-preservation of P17-tagged scFvs. Hence to our knowledge, P17 tag is the first reported peptide tag to increase the thermostability of scFvs.

As reported^33^, P17 peptide targeted multiple cargos including proteins, siRNA, and DNA-liposome complex to hepatocytes by interaction with the receptor LRP on cell surface, leading to endocytosis of cargos by hepatocytes. Indeed, P17-tagged G12-scFv was taken up by primary mouse hepatocytes more efficiently than untagged G2-scFv, as observed by immunofluorescence confocal microscopy (Supplementary Fig. S6). In this regard, besides increasing solubility and production of scFv, P17 tag can specifically augment scFv’s effect in hepatocytes by increasing intracellular concentration. In our previous study, G12-scFv targeting hepatitis B surface antigen could be endocytosed by hepatocytes and potently inhibited secretion of HBV virions^18^. Hence a stronger inhibition by P17-tagged G12-scFv than untagged version can be envisaged due to stronger antigen-binding affinity as well as more efficient uptake.

### Mechanisms by which P17 tag increases the solubility of scFv in SHuffle strain and scFv stability

Many current protein tags and peptide tags, with molecular weights of 4.5–55 kDa and 0.15–1.6 kDa, respectively, have been developed for enhancing the solubility of proteins of interest in *E. coli*^46^. Protein tags increase protein solubility by multiple mechanisms including recruitment of chaperones or functioning as intramolecular chaperones or folding nuclei to facilitate protein folding, transformation of the fusion proteins into micelle-like conformations, and enhancement of intra- and intermolecular electrostatic repulsive forces^45,46^, whereas peptide tags composed of less than 15 repetitive cationic or anionic residues do so predominantly by increasing electrostatic repulsive forces^46^.

Proteins are least soluble at the pH value equal to their isoelectric point (pI) where they do not bear net charges. Polyionic peptide tags or some protein tags can increase net charges of fusion proteins by rendering pIs more different from physiological pH^46^. This not only promotes interaction with solvent molecules but also increases the time for proper folding via stronger intra- and intermolecular repulsive electrostatic forces within a fusion protein. Such a mechanism is unlikely to apply to the P17 tag because P17-N18del-D23A/E24A, though with more net positive charges, displayed weaker solubility-enhancing ability than P17-N18del instead (Fig. 7d). At physiological pH, the P17 tag, VRC01-scFv, and G12-scFv with predicted pIs of 9.82, 8.84, and 8.35 have positive charges, but MA18/7-scFv and ADRI-scFv with pIs of 5.52 and 5.53 have negative charges (Fig. 1d). Disregarding charge accessibility, the P17 tag increases net positive charges for VRC01-scFv, and G12-scFv, but reduces overall net charges of MA18/7-scFv and ADRI-scFv. Nonetheless, the P17 tag had a weaker solubility-enhancing effect on VRC01-scFv and G12-scFv than on MA18/7-scFv and ADRI-scFv (Fig. 1d), arguing against the supercharging model^46^. Forcing P17-tagged scFv into micelle-like structures is also unlikely since SEC only revealed monomeric and dimeric forms of G12-scFv-HA-P17 (Fig. 10a). The same holds for chaperone recruitment by the P17 tag because two or three copies of P17 tags did not increase but rather weaken the solubility enhancement of a single tag copy (Fig. 4). Though it is not excluded that P17 tag residues 22 to 29 adopt an α-helical secondary structure in P17-tagged scFvs, such an α-helix is dispensable for solubility enhancement, as evidenced by point mutation and deletion mutation analysis (Figs. 5 and  6d). We also found no positive evidence for an interaction of the P17 tag with RNA, which could contribute to chaperoning^41–44^.

Higher thermostability, as well as stronger antigen affinity and virus neutralization activity of P17-tagged versus untagged G12-scFv-HA, suggest that the P17 tag promoted correct folding of scFv via an intramolecular chaperone-like activity. Two types of intramolecular chaperones are currently defined^55^. Type I intramolecular chaperones assist tertiary structure formation at an early stage of protein folding by acting as the folding nucleus or by preventing misfolding and aggregation, while type II intramolecular chaperones are not directly involved in protein folding but play key roles in the formation of quaternary structure^55^. P17 tag, monomeric on its own, did not alter the quaternary conformation of scFv (Fig. 10). Moreover, excess P17 peptide added in trans post purification did not increase thermostability of G12-scFv (Fig. 8b). All these data suggested that P17 tag functions as type I intramolecular chaperone. During folding of the scFv-P17 fusion protein, the P17 tag may interact with hydrophobic regions of folding intermediates of the scFv but also with solvent molecules through the many polar residues. This will promote protein correct folding and inhibit formation of aggregates. Indeed, reducing the number of charged residues impaired the solubility-enhancing ability of the P17 tag (Fig. 7d), similar to non-ATP hydrolyzing holdase chaperones and α-crystallin-derived peptide mini-chaperones^62–65^.

Besides solubility, stability is the other challenge for practical applications of scFv. The overall stability of two-domain scFvs including the intrinsic stability of VH and VL domains, and the stability of the hydrophobic VH–VL interface relate closely to the specific sequences of the complementary determining regions and the intrinsic stability of subdomains such as β barrels^9^. Intradomain disulfide bonds connecting two β sheets per scFv monomer are also critical for intrinsic domain stability of many scFvs, as one disulfide bond contributes 4-6 kcal/mol of free energy to protein folding^66^. This was corroborated by an ~3 °C Tm decrease of G12-scFv upon DTT treatment (Fig. 8a). Notably, the P17 tag enhanced the Tm of G12-scFv by 2.3 °C (Fig. 8b), thus increasing overall stability of scFv as efficiently as intradomain disulfide bonds. Hence most likely, the scFv stability increase by the P17 tag is as well due to type I intramolecular chaperone activity. Thereby the P17 tag may promote protein folding by increasing the unfolding energy barrier and by stabilizing the hydrophobic VH–VL interface, eventually reducing promiscuous domain-swapping to oligomerize monomer and dimer into soluble or insoluble aggregates^9,67^.

## Methods

### Ethical statement

The sera of CHB patients were obtained from Drs. Demin Yu and Xinxin Zhang from Ruijin Hospital of Shanghai JiaoTong University School of Medicine. The research use of these sera has been approved by the Ruijin Hospital Ethics Committee (EC approval No. 2016-124) and got informed consent from the donors.

### Plasmid constructs

pET28a-His-G12-scFv-HA, pET28a-His-MA18/7-scFv-HA, and pET28a-His-VRC01-scFv-HA plasmids expressing G12-scFv, MA18/7-scFv, and VRC01-scFv with N and C termini attached respectively by hexahistidine and HA tags have been described^18^. pET28a-His-ADRI-scFv-HA was constructed by replacing the G12-scFv open reading frame (ORF) of pET28a-His-G12-scFv-HA with a customized ADRI-scFv ORF^36^ via digestion with NdeI and BamHI restriction endonucleases.

To construct pET28a-His-G12-scFv-P17 and pET28a-His-MA18/7-scFv-P17 plasmids expressing G12-scFv-P17 and MA18/7-scFv-P17 proteins with the P17 tag directly fused to their termini, the complete G12-scFv-P17 and MA18/7-scFv-P17 ORFs were separately amplified by two runs of PCR. Using pET28a-His-G12-scFv-HA and pET28a-His-MA18/7-scFv-HA as the templates, the first-run PCR products were amplified separately by two pairs of primers: G12-VL-NdeI-fwd: 5’-ggcagccatatggacatcgagctgacccagag-3’ plus P17-rev1: 5’-aaacccttggtctcatcgcgccactgctttgtgttcgtagcgttggtactggactcgttcttgctaccgccacctccggat-3’ and 18/7-VL-NdeI-fwd: 5’-ggcagccatatggacattgtgatgacccagt-3’ plus P17-rev1, and used as the templates for the second run of PCR. Full-length G12-scFv-P17 and MA18/7-scFv-P17 genes were then amplified by two other pairs of primers: G12-VL-NdeI-fwd plus P17-XhoI-rev2: tggaacctcgagtcaaccagccgtattcttgaaccgcttggcttcgtctcggaaacccttggtctcatcgcg-3’ and 18/7-VL-NdeI-fwd plus P17-XhoI-rev2 and inserted into pET28a vector by digestion with NdeI and XhoI endonucleases.

To construct plasmids pET28a-His-G12-scFv-HA-P17, pET28a-His-MA18/7-scFv-HA-P17, pET28a-His-VRC01-scFv-HA-P17, and pET28a-His-ADRI-scFv-HA-P17 expressing various scFv-HA-P17 fusion proteins with a HA tag inserted between scFv and P17 tag, DNA coding for HA-P17 fusion tags was amplified by PCR using pET28a-His-G12-scFv-HA as the template and the following primers: HA-P17-BamHI-fwd: 5’-gcagcggatccggatacccctacgacgtgcccgactacgccggaggtggcggtagcaagaacgagtccagtaccaa-3’ and pET28-DraIII-rev: 5’-accgtctatcagggcgatg-3’, and then inserted respectively into pET28a-His-G12-scFv-HA, pET28a-His-MA18/7-scFv-HA, pET28a-His-VRC01-scFv-HA, and pET28a-His-ADRI-scFv-HA plasmids by digestion with BamHI and DraIII endonucleases.

To construct pET28a-His-P17-MA18/7-scFv-HA plasmid expressing P17-MA18/7-scFv-HA protein with P17 tag attaching at N terminus, P17 gene was amplified by PCR using pET28a-His-MA18/7-scFv-HA-P17 as the template and the following primers: P17-NdeI-fwd: 5’-ggcagccatatgaagaacgagtccagtacc-3’ and P17-NdeI-rev: 5’-gctgcccatatgggatccgcctccaccagccgtattcttgaac-3’, and inserted into pET28a-His-MA18/7-scFv-HA plasmid by digestion with NdeI endonuclease.

To construct pET28a-his-G12-scFv-MBP expressing G12-scFv-MBP fusion protein with MBP tag attaching at C-terminus, MBP gene released from pET28-miniDP-MBPH6 plasmid by digestion with KpnI and XbaI was used to replace HA tag gene of pET28a-His-G12-scFv-HA.

pET28a-His-MA18/7-scFv-2×P17 plasmid was constructed by ligation of BamHI-digested pET28a-His-MA18/7-scFv-P17 vector with BamHI/BglII-digested P17 gene amplified by PCR using pET28a-His-MA18/7-scFv-P17 as the template and two primers: P17-BamHI-fwd: 5’-cttacggatccggaggtggcggtagcaagaacgagtccagtacc-3’ and P17-BglII-rev: 5’-gtaagagatctaccagccgtattcttgaac-3’.

pET28a-His-MA18/7-scFv-3×P17 plasmid was then obtained by ligation of another copy of BamHI/BglII-digested P17 gene into BamHI-cleavaged pET28a-His-MA18/7-scFv-2×P17 vector. To construct pET28a-His-MA18/7-scFv-HA-P17-A25P plasmid expressing MA18/7-scFv-HA protein attached by a mutated P17 tag with alanine (A) at position 25 substituted by proline (P) at C-terminus, DNA encoding MA18/7-scFv-HA-P17-A25P protein was amplified by PCR using pET28a-His-MA18/7-scFv-HA-P17 plasmid as the template and two primers: G12-VL-NdeI-fwd and P17-A25P-XhoI-rev: 5’-tggaacctcgagtcaaccagccgtattcttgaaccgcttcggttcgtctcg-3’, and inserted into pET28a vector by digestion with NdeI and XhoI endonucleases.

To construct pET28a-His-G12-scFv-HA-P17-C8del and pET28a-His-G12-scFv-HA-P17-C15del plasmids expressing G12-scFv-HA fusion protein attached by deletion mutants of P17 tag with C-terminal 8 aa and 15 aa deleted, G12-scFv-HA-P17-C8del and G12-scFv-HA-P17-C15del genes were amplified separately by PCR using pET28a-His-G12-scFv-HA-P17 as the template and two pairs of primers: G12-VL-NdeI-fwd plus P17-C8del-XhoI-rev: 5’-tggaacctcgagtcaggcttcgtctcggaaacc-3’ and G12-VL-NdeI-fwd plus P17-C15del-XhoI-rev: 5’-tggaacctcgagtcaggtctcatcgcgccactgc-3’, and inserted into pET28a vector by digestion with NdeI and XhoI endonucleases.

To construct pET28a-His-G12-scFv-HA-P17-N8del, pET28a-His-G12-scFv-HA-P17-N14del, and pET28a-His-G12-scFv-HA-P17-N18del plasmids expressing G12-scFv-HA fusion proteins attached by deletion mutants of P17 tag with N-terminal 8 aa, 14 aa, and 18 aa deleted, G12-scFv-HA-P17-N8del, G12-scFv-HA-P17-N14del, and G12-scFv-HA-P17-N18del genes were amplified separately by PCR using pET28a-His-G12-scFv-HA-P17 as the template and three pairs of primers: P17-N8del-BamHI-fwd: 5’-gcagcggatccggaggtagcacgaacacaaagcagtggc-3’ plus pET28-DraIII-rev, P17-N14del-BamHI-fwd: 5’-gcagcggatccggaggtagccgcgatgagaccaagggtt-3’ plus pET28-DraIII-rev, and P17-N18del-BamHI-fwd: 5’-gcagcggatccggaggtagcaagggtttccgagacgaag-3’ plus pET28-DraIII-rev, cleaved by BamHI and DraIII endonucleases, and then ligated with pET28a-His-G12-scFv-HA-P17 vector digested by BamHI and DraIII enzymes.

To construct pET28a-His-G12-scFv-HA-P17-N18del-DE, pET28a-His-G12-scFv-HA-P17-N18del-KR, and pET28a-His-G12-scFv-HA-P17-N18del-DEKR plasmids expressing G12-scFv-HA fusion proteins attached by N18del mutants of P17 tag with aspartic acid (D) at position 23 and glutamic acid (E) at position 24 replaced by alanines, lysine (K) at position 26 and arginine (R) at position 27 substituted by alanines, and four residues (D, E, K and R) at positions 23, 24, 26, and 27 replaced by alanines, DNA fragments coding for point mutants of P17-N18del were amplified by PCR using pET28a-His-G12-scFv-HA-P17-N18del as the template and three pairs of primers: P17-N18del-DE-BamHI-fwd: 5’-gcagcggatccggaggtagcaagggtttccgagccgccgccaagcggttcaagaatacggctggt-3’ plus pET28-DraIII-rev, P17-N18del-KR-BamHI-fwd: 5’-gcagcggatccggaggtagcaagggtttccgagacgaagccgccgccttcaagaatacggctggt-3’ plus pET28-DraIII-rev, and P17-N18del-DEKR-BamHI-fwd: 5’-gcagcggatccggaggtagcaagggtttccgagccgccgccgccgccttcaagaatacggctggt-3’ plus pET28-DraIII-rev.

To construct pET28a-His-miniDP-HA and pET28a-His-miniDP-HA-P17 plasmids expressing truncated duck hepatitis B virus polymerase (miniDP) fusion proteins with a HA tag and double tags of HA and P17 attaching at C-terminus, miniDP gene was amplified from pET28-miniDP-MBPH6 plasmid^68^ by PCR using the primers: miniDP-NdeI-fwd: 5’-ggcagccatatggacttaccacgccta-3’ and miniDP-BglII-rev: 5’-ccaaagatctccatctgctttcttcaatc-3’, cleavaged by NdeI and BglII endonucleases, and then ligated with pET28a-His-G12-scFv-HA and pET28a-His-G12-scFv-HA-P17 vectors digested by NdeI and BamHI endonucleases.

For constructing pET28a-P17-miniDP-his plasmid expressing P17-miniDP-his protein with N terminus attached by P17 tag, P17 gene was amplified by PCR using pET-His-miniDP-HA-P17 as the template and two primers: P17-XbaI-fwd: 5’-ctagtctagaataattttgtttaactttaagaaggagatataccatgaagaacgagtccagtaccaa-3’ and P17-NcoI-rev: 5’-cacatgccatggcagatccaccagccgtattcttgaac, and used to substitute MBP gene of pET28a-MBP-miniDP-his plasmid through double digestion with XbaI and NcoI endonucleases.

The plasmids constructed in this study will be freely available for research use from the corresponding authors within 1 month of the request being made.

### Protein expression, bacteria lysis, and protein purification

*E. coli* SHuffle T7 (New England Biolabs) or BL21 (DE3) Star (ThermoFisher Scientific) strains transformed by pET28a vector-derived scFv expressing plasmids were cultured in LB media containing 0.5 mM isopropyl β-D-1-thiogalactopyranoside (IPTG) at 30 °C for 16 h to induce expression of scFv proteins. Bacteria collected by centrifugation at 8,000 g for 5 min were suspended in 10 ml lysis buffer (20 mM Tris-HCl, pH 7.5, 0.5% Triton X-100, 150 mM NaCl, and 10 mM MgCl_2_) supplemented with 5 μg/ml DNaseI, 50 μg/ml RNaseA, 1 mg/ml lysozyme, and 1× protease inhibitor cocktail solution (Roche) per gram, and lysed on ice by 12 repeats of ultrasonic disruption with 99 cycles of sonication at 80 Watt for 1 sec and break for 1 s. Ten μl bacterial lysate representing the total bacterial protein fraction was mixed with 40 μl 2× SDS-PAGE loading buffer (100 mM Tris-HCl, pH 6.8, 4% SDS, 12% glycerol, 4 mM DTT, and 0.02% bromophenol blue) for western blotting analysis of total scFv protein. After centrifugation of bacterial lysate at 12,000×*g* at 4 °C for 20 min, 10 μl clear supernatants representing soluble fraction were mixed with 40 μl 2× SDS-PAGE loading buffer for western blotting analysis of soluble scFv protein. Soluble scFv protein in the supernatant was purified by immobilized metal ion affinity chromatography (IMAC) with an Ni-nitrilotriacetic acid (Ni-NTA) superflow column (Qiagen), followed by size-exclusion chromatography on a Superdex 200 Increase 10/300 column (GE Healthcare). ScFvs were finally dialyzed against PBS and concentrated by Amicon centrifugal ultrafiltration devices (Millipore) with a molecular cutoff of 3 kDa.

### Western blotting

Proteins mixed with 2× SDS-PAGE loading buffer were separated by electrophoresis on a SDS-12.5% PAGE, and electro-transferred onto a polyvinylidene fluoride membrane in the Towbin buffer (25 mM Tris base, 192 mM glycine, pH 8.3, 0.1% SDS, and 20% methanol) at 100 V for 90 min. After blocking the membrane with TBST buffer (20 mM Tris-HCl, pH 7.4, 150 mM NaCl, and 0.1% Tween 20) containing 5% skim milk, scFv was detected by sequential incubations with 1000-fold diluted mouse monoclonal Ab against hexahistidine tag (Proteintech, catalog no. 66005-1-Ig) and 5000-fold diluted horseradish peroxidase-conjugated goat anti-mouse secondary antibody (Jackson ImmunoResearch Laboratories), followed by reaction with chemiluminescent substrates (PerkinElmer).

### SEC

Two hundred μl protein markers (catalog No: 69385, Sigma) or test scFvs diluted in PBS were loaded onto a Superdex 200 Increase 10/300 column using ÄKTA Purifier system (GE Healthcare). Proteins eluted by PBS at a flow rate of 0.5 ml/min were monitored via ultraviolet absorbance at 280-nm wavelength. According to the user manual of the column, the average distribution constant (K_av_) of test protein was determined according to the equation K_av_ = (V_e_ − V_0_)/(V_c_ − V_0_) using the parameters: elution volume (V_e_) of test protein, void volume (V_0_) and bed volume (V_c_) of the column. The calibration curve of SEC was plotted as K_av_ of protein markers versus logarithms of their molecular weights using the linear fitting program of Origin 8.0 software.

### Biolayer interferometry

Biolayer interferometry to measure Kd reflecting binding affinity of G12-scFv-HA or G12-scFv-HA-P17 to hepatitis B surface antigen (HBsAg) was performed as previously described^18^. Amine-reactive sensors were coated with 5 µg/ml CHO cell-expressed S protein in sodium acetate buffer at pH 5.0 as recommended by the manufacturer, then set to bind various concentrations of G12-scFv-HA or G12-scFv-HA-P17 protein at 37 °C for 300 s, and dissociate in PBS for another 600 s in an Octet Red96 System (Pall ForteBio). Interference signals produced by the association or dissociation of molecules were recorded and Kd was obtained with the Octet QK software package.

### Thermal shift assay

Thermal shift assays were performed as previously described^53^ with minor modifications. SYPRO Orange dye was firstly mixed with 5 μM final concentration of scFv according to user manual (ThermoFisher Scientific). An optical 96-well reaction plate (Applied Biosystems) loaded with 20 μl reaction mixture per well was mounted onto a real-time PCR instrument (QuantStudio 6 Flex, Applied Biosystems). The mixture was heated up by 0.05 °C/s from 30 to 90 °C, and the fluorescence signal in 570-nm wavelength yielded by the dye was recorded concomitantly. Melting temperature (Tm) for each scFv was determined by fitting fluorescence signal and temperature to the Boltzmann equation using Protein Thermal Shift software v1.3 (ThermoFisher Scientific).

### ELISA

The assay was done as previously described^18^. Microwell plates coated respectively with 5 μg/ml G12-scFv-HA, G12-scFv-HA-P17, milk, or 10 μg/ml MBP-G12-scFv solutions were incubated with PBS diluted sera of healthy donors (GemCell, catalog No. 100-512) and CHB patients at 37 °C for 1 h, and subsequently reacted with peroxidase-conjugated anti-S (Shanghai Kehua Bio-Engineering Company, KHB). After extensive washing, the plates were developed using 3, 3’, 5, 5’-tetramethylbenzidine (KHB) as substrate with subsequent stopping by sulphuric acid. Absorbance values at 450 nm (A450 nm) were recorded by an ELISA plate reader (Biorad).

### Production of HBV, neutralization assay, and northern blotting

These assays were performed as previously reported^69^. Briefly, HBV particles secreted by HepAD38 cells were precipitated with 7.5% PEG8,000 solution and quantitated by real-timer quantitative PCR using HBV-specific primers. HBV at a nominal multiplicity of infection of 100 vge/cell was incubated with various concentrations of scFv at room temperature for 30 min, and then was used to inoculate 1.5 × 10^5^ HepG2-NTCP cells per well grown on a collagen-coated 24-well plate in the presence of 4% PEG8,000. After 16 h, cells were extensively washed by 500 μl PBS per well for five times and cultured with DMEM containing 2.5% DMSO until harvest. At day 6 post HBV infection, hepatitis B e antigen (HBeAg) secreted by HBV-infected cells in the culture supernatants was determined by enzyme-linked immunosorbent assay (ELISA) following the user manual. Total RNA was extracted from HBV-infected cells using TRIzol reagents (ThermoFisher) according to the manufacturer’s protocol. HBV RNA was detected by northern blotting using HBV-specific ^32^P-labeled DNA probe^69^. Densitometry of hybridization signals was done using MultiGauge V2.2 (Fujifilm).

The HepG2-NTCP cell line was previously established^18^ and here authenticated by morphology, western blotting of NTCP protein, or PCR with NTCP-specific primers. HepAD38 cell line was provided by Dr. Christoph Seeger (Fox Chase Cancer Center, Philadelphia, USA) and authenticated by morphology and PCR with HBV-specific primers.

### Immunofluorescence microscopy

The assay was conducted as previously reported^18^. Primary mouse hepatocytes grown on collagen-coated coverslips were fixed with 4% paraformaldehyde and permeabilized with 0.25% Triton X-100. After blocking with 3% bovine serum albumin (fraction V, Sigma) for 10 min, cells were incubated with the respective primary antibodies followed by Alexa Fluor 488 or Cy3 conjugated secondary antibodies. Nuclear DNA was stained with 4’, 6-diamidino-2-phenylindole (Sigma). Cells were visualized by a laser confocal scanning microscope (Leica TCS SP8). Image processing was done by Las AF Lite 2.6 software (Leica).

### Statistics and reproducibility

Results acquired by three independent experiments were presented as average values ± standard deviations. Statistical significance of differences between two experimental groups was assessed one-way analysis of variance as implemented in Origin 8.0 software. Fifty percent inhibitory concentrations of scFvs were also calculated by Origin 8.0 software.

### Reporting summary

Further information on research design is available in the Nature Research Reporting Summary linked to this article.

## Supplementary information

Supplementary information

Peer Review File

Reporting Summary

## Data Availability

Source data are provided with this paper.

## Source data

Source data
