# Supplementary material for: A 33-residue peptide tag increases solubility and stability of Escherichia coli produced single-chain antibody fragments
Source: Nat Commun. 2022 Aug 8;13:4614. doi: 10.1038/s41467-022-32423-9 (PMC9359998; doi:10.1038/s41467-022-32423-9)
Supplement: Supplementary file 1 — Supplementary information [file 41467_2022_32423_MOESM1_ESM.pdf]

Supplementary Figures and Figure Legends

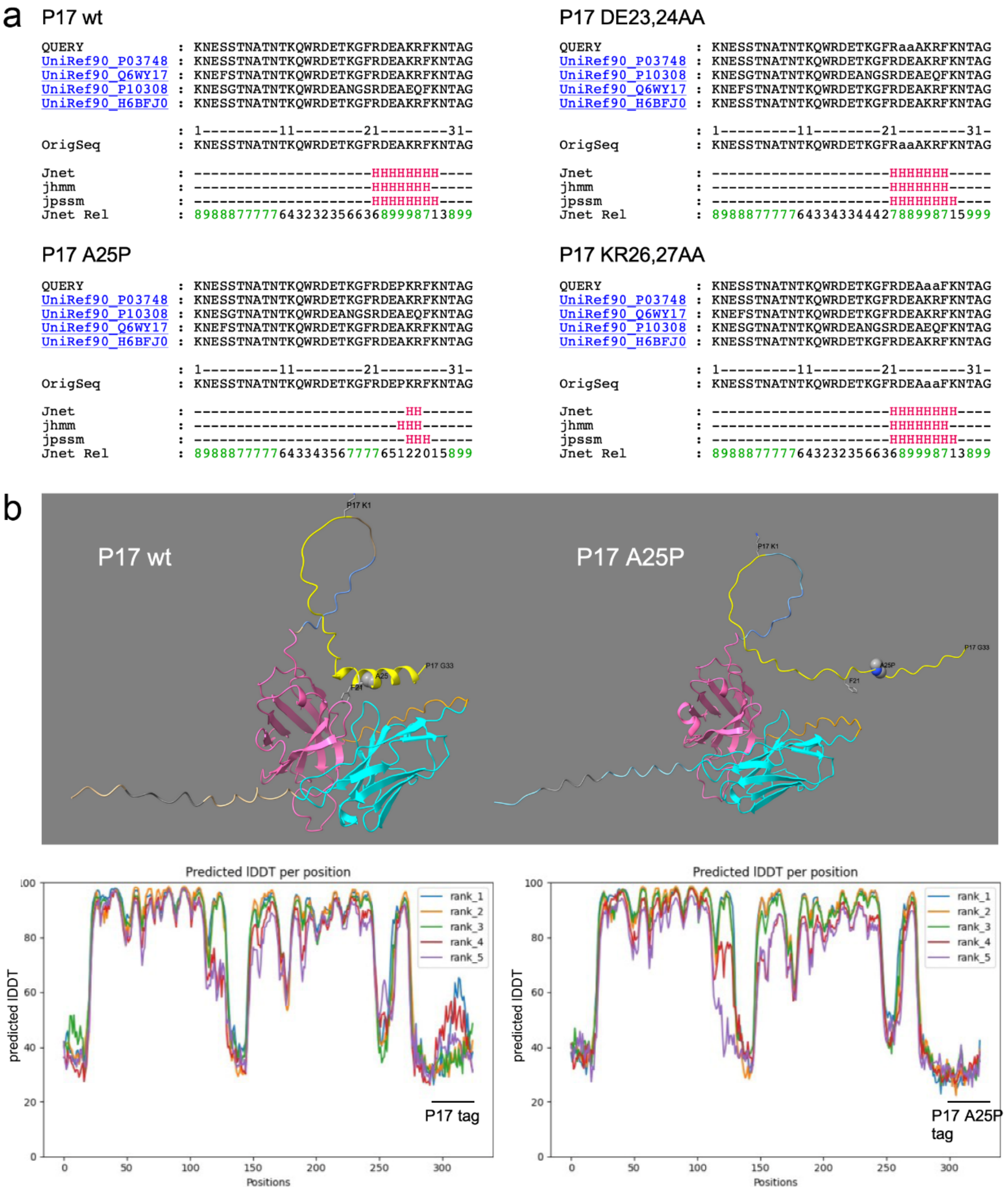

Supplementary Figure 1 Moderate predicted α-helix propensity for P17 tag residues R22-K29 but not the rest of the sequence. In addition to other secondary structure predictions we here used JPred4[1] to estimate the α-helix propensity within the P17 tag and its A25P, D23E24AA and K26R27AA variants. Furthermore, we applied the protein folding program AlphaFold2[2] as implemented in AlphaFold2.ipynb on Google Colab, to predict the 3D structure of the complete MA18/7-scFv-HA-P17 protein and its P17 A25P variant. **a** Jpred4 predictions for P17 wild-type and variant

peptides. UniRef90 numbers[3] refer to sequence clusters sharing at least 90% identity; all are derived from phage T7 gp17 gene products. Coiled coil and buried residue predictions gave no significant results and are not shown. Line Jnet refers to the consensus prediction; Jnet Rel to the confidence score from 0-9, with higher values indicating higher confidence. Note that the sequence R22-K29 is the only one giving a high confidence α-helix propensity; α-helix propensity drops drastically by the A25P mutation but remains high in the D23E24AA, K26R27AA and variants. **b** Highest ranking AlphaFold2 models for MA18/7-scFv-HA-P17 (left) and its P17-A25P variant. VL and VH chains are depicted in cyan and magenta, the connecting linker in light brown, and the P17 sequence (fom K1 to G33) in yellow, preceded by the HA tag in blue; A25 and the P residue in variant A25P are shown as spheres. The lower graphs show the predicted Local Distance Difference Test (plDDT) scores; values >90, 70-90 and 50-70 indicate modeling with high, good and low accuracy. Accordingly, confidence in the α-helix prediction for the wild-type P17 tag is already low, and completely abolished by the P17 A25P mutation.

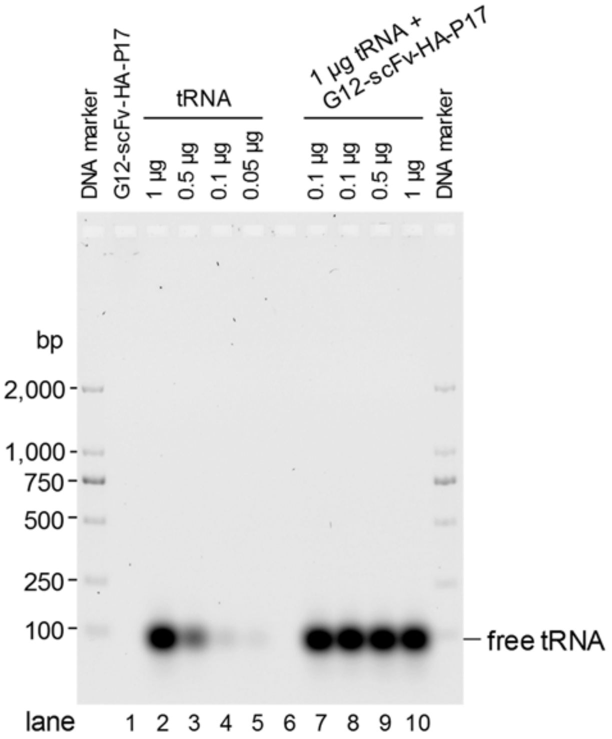

Supplementary Figure 2 G12-scFv-HA-P17 protein did not bind tRNA. One μg purified G12-scFv-HA-P17 protein (Lane 1), various amounts of tRNA (Lanes 2 to 5), and 1 μg tRNA incubated with various amounts of purified G12-scFv-HA-P17 protein at 37 °C for 30 min (Lanes 7 to 10) were separately electrophoresed on a TAE-1.2% agarose gel and stained by ethidium bromide. The result of one representative experiment was shown.

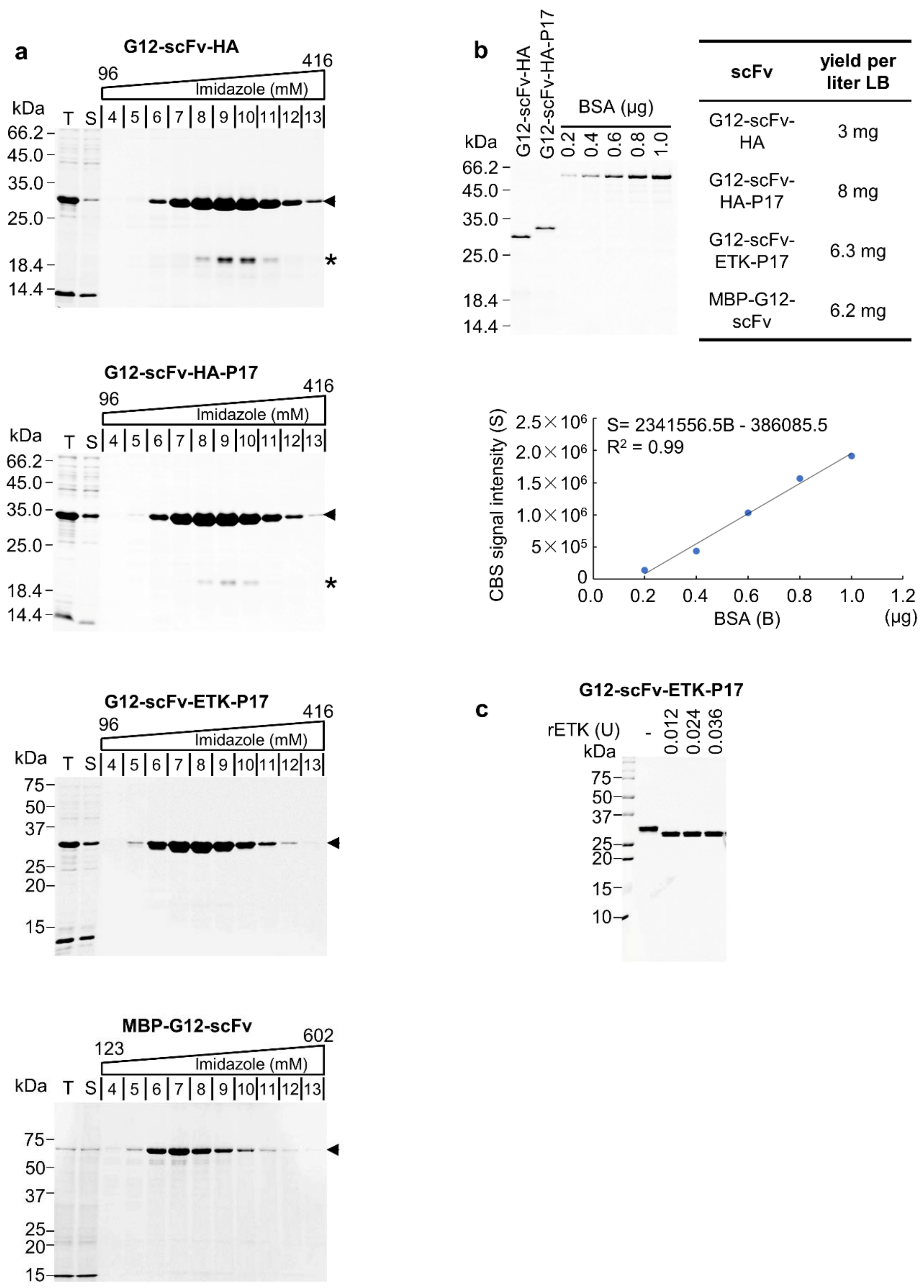

scFv	yield per liter LB
G12-scFv-HA	3 mg
G12-scFv-HA-P17	8 mg
G12-scFv-ETK-P17	6.3 mg
MBP-G12-scFv	6.2 mg

Supplementary Figure 3 Expression and purification of recombinant G12-scFv-HA, G12-scFv-HA-P17, and G12-scFv-ETK-P17 proteins and efficient removal of P17 tag by enterokinase digestion. a Purification of hexa-histidine tagged G12-scFv-HA, G12-scFv-HA-P17, G12-scFv-ETK-P17, and MBP-G12-scFv proteins through

Ni-NTA chromatography. Total lysates (T) and their soluble fractions (S) from *E. coli* SHuffle T7 strains cultured in Luria-Bertani (LB) media containing 0.5 mM IPTG were prepared as mentioned in Methods. After loading S fractions onto Ni-NTA columns, G12-scFv-HA, G12-scFv-HA-P17, and G12-scFv-ETK-P17 proteins bound by Ni-NTA were eluted with a linear concentration gradient (96 mM-416 mM or 123-602 mM) of imidazole. T, S, and eluted scFvs were analyzed by SDS-12.5% PAGE and Coomassie-Blue staining (CBS). The result of one representative experiment was shown. The positions of ~30 kDa G12-scFv-HA, G12-scFv-HA-P17, G12-scFv-ETK-P17, and 69 kDa MBP-G12-scFv proteins are indicated by arrowheads. *, degraded fragment. **b** Quantitation of purified G12-scFv-HA and G12-scFv-HA-P17 proteins using bovine serum albumin (BSA) as the standard and soluble yields of G12-scFv-HA, G12-scFv-HA-P17, G12-scFv-ETK-P17, and MBP-G12-scFv proteins per liter LB culture. One μl final products of G12-scFv-HA and G12-scFv-HA-P17 proteins and various amounts of BSA separated by SDS-12.5% PAGE were stained by Coomassie-Blue (upper panel). Calibration curve and equation of BSA amount and its CBS signal intensity quantitated by MultiGauge V2.2 software were obtained using linear fitting program of Origin 8.0 software (bottom panel). The soluble yields per liter LB culture for four scFvs (upper panel) were assessed by determining their final concentrations according to the calibration curve. **c** Efficient removal of the P17 tag from G12-scFv-ETK-P17 protein by digestion with enterokinase. Twenty-two μg G12-scFv-ETK-P17 protein with a enterokinase digestion site (DDDDK) inserted between G12-scFv and the P17 tag was digested by 0.012, 0.024, and 0.026 units of enterokinase (Yeasen, Shanghai; one unit is defined as the amount of enzyme that will cleave 500 μg of fusion protein in 12-16 hours at 25°C, in a buffer containing 25 mM Tris-HCl, pH 8.0), respectively, at 25°C for 16 h in a 50-μl reaction volume. After centrifugation at 20,000 g for 30 min at 4 °C, 2.5 μl of supernatant from each digestion mixture was analyzed by SDS-12.5% PAGE and subsequent CBS. The result of one representative experiment was shown.

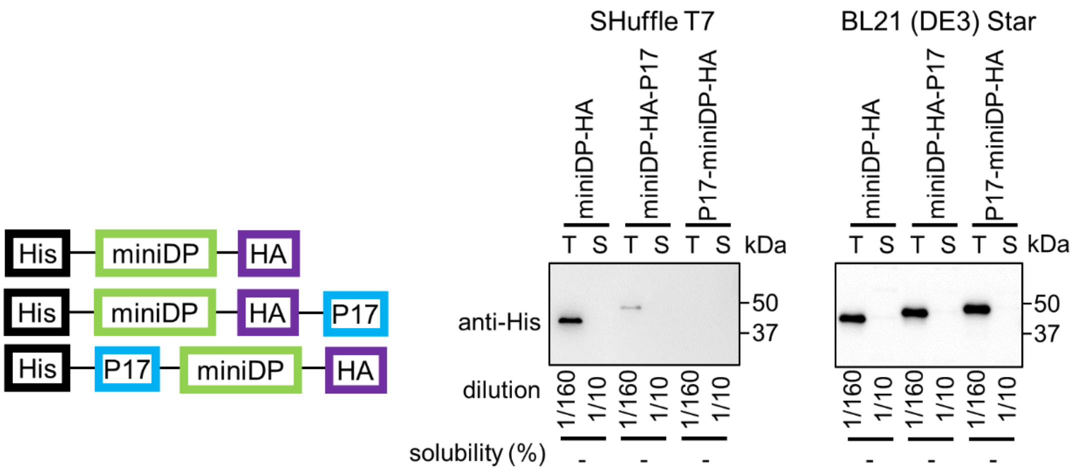

Supplementary Figure 4 P17 tag did not increase the solubility of truncated duck hepatitis B virus polymerase (miniDP) in *E. coli*. Left panel: schematic diagrams of His-miniDP-HA fusion protein and its derivatives (His-miniDP-HA-P17 and His-P17-miniDP-HA) with P17 tag attached at N and C termini. After diluted by 1/160 and 1/10, total (T) and soluble (S) fractions of His-miniDP-HA fusion protein and its p17 tagged derivatives in *E. coli* SHuffle T7 (Middle panel) and BL21(DE3) Star (Right panel) strains were detected by Western blotting. Solubility was determined according to the description in Fig. 1. -, below detection limit. The result of one representative experiment was shown.

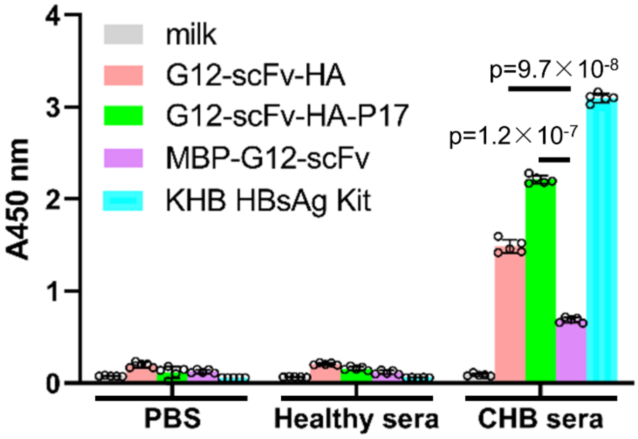

Supplementary Figure 5 Enzyme linked immunosorbent assay (ELISA) to test binding affinity of G12-scFv-HA, G12-scFv-HA-P17, and MBP-G12-scFv proteins. The pooled sera of chronic hepatitis B patients and healthy donors were confirmed to be positive and negative for hepatitis B surface antigen (HBsAg) by a commercial KHB ELISA kit, and then reacted with five repetitive microtiter wells coated with 5 μg/ml solutions of G12-scFv-HA and G12-scFv-HA-P17, and 10 μg/ml MBP-G12-scFv solution, respectively. Data are presented as mean values ± SD. Statistical significance of differences between two experimental groups was assessed one-way analysis of variance.

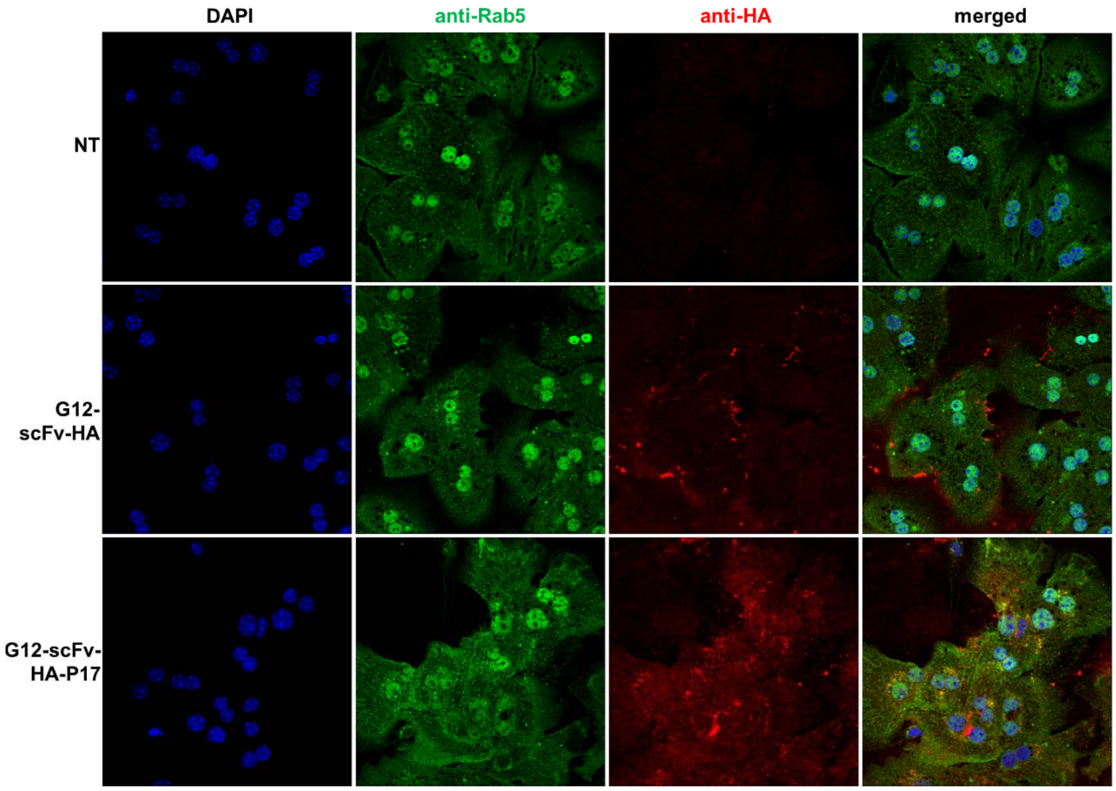

Supplementary Figure 6 P17 tag enhanced endocytosis of G12-scFv by primary mouse hepatocytes. Primary mouse hepatocytes grown on collagen-coated coverslips were treated with 10 μg/ml G12-scFv-HA and G12-scFv-HA-P17 proteins for 24h or not. After fixed by 4% para-formaldehyde and permeabilized with 0.25% Triton X-100, cells were incubated with 300-fold diluted rat anti-HA and rabbit anti-Rab5 primary antibodies, followed by 300-fold diluted Cy3- and Alexa Fluor 488-conjugated secondary antibodies. Signals of Rab5 (in green), scFv (in red), and nuclear DNA (in blue) were visualized by laser confocal microscopy. The result of one representative experiment was shown.

References

1. Drozdetskiy A, Cole C, Procter J, Barton GJ. JPred4: a protein secondary structure prediction server. *Nucleic Acids Res* **43**, W389-394 (2015).
2. Jumper J, *et al.* Highly accurate protein structure prediction with AlphaFold. *Nature* **596**, 583-589 (2021).
3. Suzek BE, Huang H, McGarvey P, Mazumder R, Wu CH. UniRef: comprehensive and non-redundant UniProt reference clusters. *Bioinformatics* **23**, 1282-1288 (2007).
