# Supplementary material for: A 33-residue peptide tag increases solubility and stability of Escherichia coli produced single-chain antibody fragments
Source: Nat Commun. 2022 Aug 8;13:4614. doi: 10.1038/s41467-022-32423-9 (PMC9359998; doi:10.1038/s41467-022-32423-9)
Supplement: Supplementary file 3 — Reporting Summary [file 41467_2022_32423_MOESM3_ESM.pdf]

nature portfolio

Corresponding author(s): Yong-Xiang Wang, Shijie Chen

Last updated by author(s): Jul 21, 2022

Reporting Summary

Nature Portfolio wishes to improve the reproducibility of the work that we publish. This form provides structure for consistency and transparency in reporting. For further information on Nature Portfolio policies, see our Editorial Policies and the Editorial Policy Checklist.

Statistics

For all statistical analyses, confirm that the following items are present in the figure legend, table legend, main text, or Methods section.

n/a	Confirmed	
☐	☒	The exact sample size (*n*) for each experimental group/condition, given as a discrete number and unit of measurement
☐	☒	A statement on whether measurements were taken from distinct samples or whether the same sample was measured repeatedly
☐	☒	The statistical test(s) used AND whether they are one- or two-sided<br>*Only common tests should be described solely by name; describe more complex techniques in the Methods section.*
☒	☐	A description of all covariates tested
☒	☐	A description of any assumptions or corrections, such as tests of normality and adjustment for multiple comparisons
☐	☒	A full description of the statistical parameters including central tendency (e.g. means) or other basic estimates (e.g. regression coefficient) AND variation (e.g. standard deviation) or associated estimates of uncertainty (e.g. confidence intervals)
☐	☒	For null hypothesis testing, the test statistic (e.g. *F*, *t*, *r*) with confidence intervals, effect sizes, degrees of freedom and *P* value noted<br>*Give P values as exact values whenever suitable.*
☒	☐	For Bayesian analysis, information on the choice of priors and Markov chain Monte Carlo settings
☒	☐	For hierarchical and complex designs, identification of the appropriate level for tests and full reporting of outcomes
☒	☐	Estimates of effect sizes (e.g. Cohen's *d*, Pearson's *r*), indicating how they were calculated

Our web collection on statistics for biologists contains articles on many of the points above.

Software and code

Policy information about availability of computer code

Data collection	none
Data analysis	Las AF Lite software (Leica, version 2.6); Origin software OriginLab (version 8.0); MultiGauge (Fujifilm Life Science, version 2.2); Protein Thermal Shift software (ThermoFisher Scientific, version 1.3)

For manuscripts utilizing custom algorithms or software that are central to the research but not yet described in published literature, software must be made available to editors and reviewers. We strongly encourage code deposition in a community repository (e.g. GitHub). See the Nature Portfolio guidelines for submitting code & software for further information.

Data

Policy information about availability of data

All manuscripts must include a data availability statement. This statement should provide the following information, where applicable:

- Accession codes, unique identifiers, or web links for publicly available datasets
- A description of any restrictions on data availability
- For clinical datasets or third party data, please ensure that the statement adheres to our policy

Data availability. The original data and figures for Figs. 1a-c, 2b, 2c, 3a, 3b, 4a, 4b, 5b, 5c, 6b, 6c, 6e, 6f, 7b, 7c, 8, 9, 10a, 10b and Supplementary Fig. 3, 4, 5, and 6 are provided in a Source Data file.

Field-specific reporting

Please select the one below that is the best fit for your research. If you are not sure, read the appropriate sections before making your selection.

☒ Life sciences ☐ Behavioural & social sciences ☐ Ecological, evolutionary & environmental sciences

For a reference copy of the document with all sections, see nature.com/documents/nr-reporting-summary-flat.pdf

Life sciences study design

All studies must disclose on these points even when the disclosure is negative.

Sample size	Four scFvs with their variable regions encoded by different subgroups of immunoglobulin heavy and light chain variable gene cluster were used to verify the solubility-enhancing effect of P17 tag. Heavy chains are encoded by a single gene cluster (IGH), light chains by the κ cluster (IGK) and the λ cluster (IGL). Sequence analysis of the four scFvs using online International ImMunoGeneTics Information System (http://www.imgt.org/) revealed that their variable regions are encoded by different subgroups of heavy (IGHV) and light chain variable gene clusters (IGKV, and IGLV). Accordingly, the variable regions of MA18/7-scFv are encoded by IGHV3 and IGKV6; those of G12-scFv by IGHV4 and IGKV3; those of VRC01-scFv by IGHV1 and IGKV1; and those of ADRI-scFv by IGHV3 and IGLV3. Although these scFvs vary considerably in sequence and their intrinsic solubility properties in E. coli, attaching the P17 tag improved the solubility of all by at least 2.4-fold and up to 11.6-fold (Fig. 1 and 2).
Data exclusions	none
Replication	Each experiment was repeated three to four times to test the solubility of scFvs attached by wild type P17 tag or its mutants, to determine melting temperature of scFvs and 50% inhibitory concentrations of scFvs to suppress hepatitis B virus infection of HepG2-NTCP cells. All the attempts at replication under our experimental conditions described in Methods section were successful.
Randomization	Not relevant for this study: only one variable is tested in each experiment
Blinding	Not relevant for this study: only one variable is tested in each experiment

Reporting for specific materials, systems and methods

We require information from authors about some types of materials, experimental systems and methods used in many studies. Here, indicate whether each material, system or method listed is relevant to your study. If you are not sure if a list item applies to your research, read the appropriate section before selecting a response.

Materials & experimental systems

n/a	Involved in the study	
☐	☒	Antibodies
☐	☒	Eukaryotic cell lines
☒	☐	Palaeontology and archaeology
☒	☐	Animals and other organisms
☒	☐	Human research participants
☒	☐	Clinical data
☒	☐	Dual use research of concern

Methods

n/a	Involved in the study	
☒	☐	ChIP-seq
☒	☐	Flow cytometry
☒	☐	MRI-based neuroimaging

Antibodies

Antibodies used	Mouse monoclonal Ab against hexa-histidine tag (Proteintech, catalog no. 66005-1-Ig, clone no. 1B7G5); Rat monoclonal Ab targeting HA tag (Roche, catalog no. 11867423001, clone no. 3F10); Rabbit polyclonal Ab against Rab5 (Proteintech, catalog no. 11947-1-AP); HRP conjugated goat anti-mouse IgG (Jackson ImmunoResearch Laboratories, catalog no. 115-035-003); Alexa Fluor 488-conjugated goat anti-rabbit IgG (Jackson ImmunoResearch Laboratories, catalog no. 111-545-003); Cy3-conjugated goat anti-rat IgG (Jackson ImmunoResearch Laboratories, catalog no. 112-165-003).
Validation	According to product information of mouse monoclonal Ab against hexa-histidine tag (https://www.ptglab.com/products/His-Tag-Antibody-66005-1-Ig.htm), anti-His Ab is suitable for Western blotting and other immunological detections, and has been validated by 346 publications.<br>According to manufacturer's information (https://www.ptglab.com/products/RAB5A-Antibody-11947-1-AP.htm), anti-Rab5 is suitable for the immunofluorescence experiment and other immunological detections, and has been validated by 19 publications.<br><br>According to product information (https://www.sigmaaldrich.cn/CN/en/product/roche/roahaha?context=product), rat monoclonal anti-HA is suitable for multiple immunological detections including immunofluorescence staining, which was also validated by our previous publication: IFN-inducible MX2 is a host restriction factor of hepatitis B virus replication. Journal of Hepatology. 2020, 72: 865-876.

HRP conjugated anti-mouse IgG, Alexa Fluor 488-conjugated anti-rabbit IgG and Cy3-conjugated anti-rat IgG are separately suitable for Western blotting and immunofluorescence experiment according to user manuals of Jackson ImmunoResearch Laboratories, and were validated by our previous publications:
1) IFN-inducible MX2 is a host restriction factor of hepatitis B virus replication. Journal of Hepatology. 2020, 72: 865-876.
2) An E. coli-produced single-chain variable fragment (scFv) targeting hepatitis B virus surface protein potently inhibited virion secretion, Antiviral Research, 2019, 162: 118-129.

Eukaryotic cell lines

Policy information about cell lines

Cell line source(s)	HepG2-NTCP cell line was established by Laboratory of Medical Molecular Virology, Fudan University as described previously (Antiviral Research, 2019, 162: 118-129); HepAD38 cell line was provided by Dr. Christoph Seeger (Fox Chase Cancer Center, Philadelphia, USA).
Authentication	HepG2-NTCP cell line was authenticated by morphology, Western blotting, or PCR with NTCP specific primers. HepAD38 cell line was authenticated by morphology, PCR with HBV specific primers.
Mycoplasma contamination	All the cell lines were mycoplasma negative verified by PlasmoTest™-Mycoplasma Detection Kit (InvivoGen catalog no. rep-pt1)
Commonly misidentified lines (See ICLAC register)	None
