# Supplementary material for: A 33-residue peptide tag increases solubility and stability of Escherichia coli produced single-chain antibody fragments
Source: Nat Commun. 2022 Aug 8;13:4614. doi: 10.1038/s41467-022-32423-9 (PMC9359998; doi:10.1038/s41467-022-32423-9)
Supplement: Supplementary file 2 — Peer Review File [file 41467_2022_32423_MOESM2_ESM.pdf]

nature portfolio

# Peer Review File

## A 33-residue peptide tag potently increases solubility and stability of E. coli- produced single-chain antibody fragments

**Open Access** This file is licensed under a Creative Commons Attribution 4.0 International License, which permits use, sharing, adaptation, distribution and reproduction in any medium or format, as long as you give appropriate credit to the original author(s) and the source, provide a link to the Creative Commons license, and indicate if changes were made. In the cases where the authors are anonymous, such as is the case for the reports of anonymous peer reviewers, author attribution should be to 'Anonymous Referee' followed by a clear attribution to the source work. The images or other third party material in this file are included in the article's Creative Commons license, unless indicated otherwise in a credit line to the material. If material is not included in the article's Creative Commons license and your intended use is not permitted by statutory regulation or exceeds the permitted use, you will need to obtain permission directly from the copyright holder. To view a copy of this license, visit http://creativecommons.org/licenses/by/4.0/.

Reviewers' comments:

Reviewer #1 (Remarks to the Author):

Review of Wang et al. submitted to Nature Communications.

"A 33-residue peptide tag potently increases solubility and stability of E. coli produced single-chain antibody fragments"

In this manuscript, the authors present the use of a small peptide tag named P17 in improving when fused, the expression, stability and antigen binding capabilities of scFv expressed in SHuffle cells. The manuscript is clearly written, the data presented is informative and the conclusions reached are supported. The only major issue is the use of C-terminally fused MBP in Figure 3. Beyond that, there does not seem to be any major issues with the manuscript and few small corrections and edits, it should be ready for publication.

Major Comment:

- It is well documented that the capacity to improve folding by MBP occurs mostly when fused N-terminally but not C-terminally. It is assumed that MBP must fold first when exiting the ribosome exit tunnel, in order to facilitate the folding of the emerging nascent chain it is fused to. Thus the conclusions reached in Figure 3 can not be supported, until the experiment is repeated with an N-temrinal fusion of MBP to the cargo protein. We strongly suggest this experiment to be repeated or the data removed.

Minor Points:

- Line 81: The authors state that the rapid clearance of scFv is advantageous, but I have read many times that it's the opposite. That IgG is retained longer in the body and has longer lasting effect. Authors should clarify that in certain conditions, rapid clearing could be preferred but is not necessarily an advantage.

- Line 107, 113, 132 and 174: Several times in the manuscript, gene names such trxB and gor along with in vitro and in vivo were not italicized. Please go over the manuscript and correct.

- Line 109: Isn't DsbA a thiol disulfide-bond oxidase instead of a sulfhydryl oxidase?

- Line 130: "…T7 phage tail can target…X,Y,Z…" to what?

- Line 140: What is a "sequential" property? Do the authors mean the property of the peptide coming from the sequence? What is the difference between the sequence of a peptide and the structure it forms? Please clarify

- Line 170: Are the two decimal points significant and reproducible? If not please round up.

- Line 195: Authors claim that HA tag did not contribute to folding. But if one compares in Fig 2b the lanes 4 to 6, one can clearly see a negative effect of having HA. Please correct that HA does have an effect.

- Line 219: The description “P17 tag may be acquired” is confusing. Please clarify and re-word. Perhaps, “may be attributed to…”

- Line 236: The authors claim to disrupt the helix by inserting a proline but have not demonstrated that the proline substitution had any effect on the folding of P17. Please soften the conclusion to indicate that the proline substitution did not have any observable effect, potentially suggesting that the exact nature of the helix does not play a significant role, according to this single mutant construct.

- Line 249: Why did it authors truncate by 8 and 15 aa? Please explain

- Line 249: Who is “they”?

- Line 301: The authors claim that P17 stablizes scFv by “inhibiting exposure to hydrophobic regions” but at this stage this is very speculitave and they finish the paragraph by stating that P17 promotes protein folding. Please clarify, whether its protein folding or inhibiting exposure to hydrophobic regions or both and what the evidence for these claims are and re-word.

- Line 311: which small surface protein?

- Line 325: should be “self-assembly into dimers”

- Line 331: aren’t the ranges from 0.137 kD to 670 kD? There are two peaks, one at 13.7 and the other 0.137.

- Line 404: the authors claim that there is no need to remove the P17 tag, but do not show any evidence of this. Does the cargo remain soluble after the removal of P17? If as the authors claim, P17 inhibits exposure to hydrophobic regions, will it remain to do so after the folding of scFv? Also, the authors state that “there is no need to cleave off the P17 tag” but I argue that choice is upto the user and the needs of the experiment. The authors should clarify that the user has the choice to retain the tag, should they want to and that scFv-P17 fusion can still be used for binding studies, should they want to.

- Line 458: “P17 tag could also may be excluded”

- Figure 1b/c: Are the two decimal points in % solubility significant? If not, round up.

- Figure 1d: The constructs should be in order of solubility

- Figure 5: More features of the P17 tag should be shown, such as residue numbers, charged residues, kD and pI of the tag and other secondary structures such as helices and strands if any and the mutant proline should be red as in Fig 7A. It might also be worth showing the predicted structure of the tag from Alphafold and or from a real structure, if any.

- Finally, looking at the first and last residues of the P17-tag, is it possible for the authors to think of NEST-TAG instead of P17 tag? :)

Reviewer #2 (Remarks to the Author):

Reviewer's Comments

In the manuscript 'A 33-residue peptide tag potently increases solubility and stability of E. coli produced single-chain antibody fragments', the authors describe the role of a short peptide P17 tag for the solubility of multiple scFvs produced in E. coli without oligomerization or monomer-to-dimer equilibrium. The P17 tag elevated the thermostability of scFv as efficiently as intra-domain disulfide bond. Solubility-enhancing effect of P17 tag was mainly attributed to its hydrophilic sequence especially ionic residues, but independent of the proposed α-helix secondary structure. The authors also suggested that P17 tag is a type I intramolecular chaperone to promote the solubility and folding of scFv and to inhibit aggregate formation, benefiting the research, production, and application of scFv.

While the paper interesting in exploiting intramolecular chaperone function for the folding of svFv, the main conclusions are based on only preliminary data, and requires further extensive analyses as suggested below.

Major Points:

1. A major criticism on the claim for promoting the solubility. The soluble fraction was defined throughout the text as the fraction that remained soluble after addition of SDS buffer to the 'clear supernatant' (M&M p21, line 621). Traditionally, the 'clear supernatant' (in this paper) has been defined as 'soluble' fraction in most of prior arts to address to their abilities to enhance their solubility and thus the productivity of the protein of interest.

2. Because of a rather narrow definition of solubility adopted in this m/s, it is really impossible to calculate the overall yield of the scFv proteins. In fact, the overall yield was not mentioned throughout the text, which should be an integral part for adopting the microbial production with a view to address to the cost and yield (Intro. p3, line 84; Discussion. P12, line 351).

3. The data is lacking on the removal of the P17 tag after purification. The cleavage and the final yield is an essential part of the paper, especially because of potential immunogenicity of the p17 tag in the fusion construct. The authors claim - "Therefore, there is no need to cleave off the P17 tag after

purification of scFv-P17 fusion protein” (Discussion. P14, line 405)- cannot be justified from the view of regulatory process. Otherwise, the authors should provide an evidence that the P17 tag is not immunogenic.

4. A major debate arises on the mode of action of the P17 tag as an intramolecular chaperon. Specifically, the conclusion that the action is independent of the proposed α-helix secondary structure (Abst. 02, line 43) is based on a limited mutagenesis on the sequence of P17 (as shown in Fig 6 & 7), and a skewed view that chaperone is proprietary of proteins. The present reviewer executed the heptad repeat helical wheel depiction of P17, which reveals a ‘sidedness’ of positive charge of the alpha-helix, which can interact with negatively charged molecule, such as tRNAs of high molecular abundance inside the bacterial cytoplasm. (Kwon et al, Int J Mol Sci. 2018 Oct 3;19(10):3016) providing chaperone function. It merits further analyses on potential transition of P17 tag from the disorder into alpha-helix induced by tRNA interaction, and whether the proposed intramolecular chaperone function of P17 tag (Abst. P2, line 41) is actually mediated by tRNA interaction (refer to recent articles on Chaperna (RNA as chaperones) on the folding of target proteins; Nanomedicine. 2021 Oct;37:102438.; Biomaterials. 2021 Feb;269:120650; Biotechnol Bioeng. 2019 Mar;116(3):490-502).

More specifically, further mutations on positively charged residues (single, or multiple mutations) in Fig 6 should be analyzed before suggesting the mechanism of the chaperone function; whether chaperone function is mediated by tag alone (as suggested by the authors) or by interaction with RNA molecules. Does the fusion protein co-purify with RNA molecules? (refer to Biomaterials. 2021 Feb;269:120650).

Minor points:

1. Describe how was the signal quantified. Densitometric scanning of the gel? (p6 lines 159-161; p7 line 185)

2. “Direct or indirect fusion” (p7, line 203)? The meaning of ‘indirect fusion’ is unclear.

3. “Promoting protein folding” (p11, line 304)? May discuss on the possibility of increasing the stability of the folded protein & resisting the protein unfolding.

4. 2-fold difference in binding affinity (p11, lines 313-314): Is it significant?

5. “Type 1 vs type 2 intramolecular chaperone” (p16, lines 465-467). The distinction is not rigorous in the area of protein folding and chaperone. Usually, the same chaperone provides the quality control of the client proteins by assisting folding and assembly cooperatively (Science. 2017 Dec 8;358(6368):1272-1278; Biomaterials. 2021 Feb;269:120650).

6. “non-ATP hydrolyzing chaperones” (p17, lines 482-483). Better described as foldase (ATP-dependent) and holdase (ATP-independent) type chaperones (Nat Commun. 2021 Feb 8;12(1):851).

7. Fig 2b. With respect to comparing C-terminal vs N-terminal fusion, additional data required comparing MA18/7-P17 and P17-MA18/7.

8. Fig 5. The mutation Ala into Pro is at the distal end of P17, and is not expected to influence the helix formation strongly. Mutation on the positively charged aa in heptad repeat may better address to the mechanism of chaperone action (Major point 4).

Reviewer #3 (Remarks to the Author):

The authors reported that a 33-residue peptide named P17 tag potently increased solubility of multiple scFvs produced in E. coli SHuffle strain, especially if attached at the carboxyl terminus. Solubility-enhancing effect of P17 tag was mainly attributed to its hydrophilic sequence especially ionic residues, but independent of the proposed α-helix secondary structure. Remarkably, P17 tag elevated thermostability of scFv as efficiently as intra-domain disulfide bonds. Moreover, P17-tagged G12-scFv targeting surface protein of hepatitis B virus showed over two-fold higher antigen-binding affinity and virus-neutralizing activity than untagged version. These results is interesting and have potential significance to the related readers. Other comments were as followed.

1) There are many scFv publications focused on scFv cloning, expression, purification, characterization, especially on solubility. So the novelty of this article is not higher enough.

2) There are some publications have studied the MBP, GST, Trx, et al tag for the fusion to improve the expression of scFv, also they get the good results. So why the author to select this P17 tag? The author compared P17 to MBP. Have you compare to other tag?

3) E.coli BL21 (DE3) is a usually used host cell for expression, but the solubility is not good in BL21 this paper. SHuffle T7 is good for expression as in Fig.1. So if SHuffle T7 has the potential to express the scFv in large scale?

4) In Fig.2. HA tag seems to have a negative effect than only P17, so only His tag can purify this fusion protein efficently?

5) In Fig.4, only one P17 tag seems better. why two or three tags together is not good?

6) In Fig.7, the authors mutanted 4 amino acids to A, why only select this four?

7) There are many important issues to be addressed for scFv, such as specificity, stability,affinity, solubility, et al. So the author should improve these issues.

We thank the reviewers for their insightful comments. Below we respond point-by-point to all their critiques and suggestions which led us to various new experiments. Furthermore, we edited the text as a whole to improve readability and minimize chances for misunderstandings.

**Reviewer #1**

In this manuscript, the authors present the use of a small peptide tag named P17 in improving when fused, the expression, stability and antigen binding capabilities of scFv expressed in SHuffle cells. The manuscript is clearly written, the data presented is informative and the conclusions reached are supported.

The only major issue is the use of C-terminally fused MBP in Figure 3. Beyond that, there does not seem to be any major issues with the manuscript and few small corrections and edits, it should be ready for publication.

**Response (R):** We are grateful to the reviewer for the positive comments on our work and manuscript. The one major concern and the various minor point suggestions for editorial improvements are addressed below.

**Major Comments:**

1. -It is well documented that the capacity to improve folding by MBP occurs mostly when fused N-terminally but not C-terminally. It is assumed that MBP must fold first when exiting the ribosome exit tunnel, in order to facilitate the folding of the emerging nascent chain it is fused to. Thus the conclusions reached in Figure 3 can not be supported, until the experiment is repeated with an N-terminal fusion of MBP to the cargo protein. We strongly suggest this experiment to be repeated or the data removed.

R1: Our choice of tag positions was largely based on a comprehensive study on the impact of common protein tags including MBP, GST, TRX, GFP, or DHFR and their N- vs. C-terminal positions on the solubility of numerous *E. coli* expressed proteins, including of mammalian origin (Dyson et al. 2004. Production of soluble mammalian proteins in Escherichia coli: identification of protein features that correlate with successful expression. BMC Biotechnol. , 4: 32). The authors concluded that MBP was effective both as an N-terminal and a C-terminal tag: "MBP-H10 was the most effective tag at the C-terminus to promote protein solubility with an average construct full-length soluble yield of 5.0 mg/l, which compares well with an average of 5.8 mg/l when this tag is fused at the N-terminus. The order of C-terminal tags to promote soluble expression was similar for total expression: MBP-H10 > GST-H10 > V5-H6 > Dhfr [murine]-H10 ~ GFP-H10 ~ Trx-H10 > H10 ~ DHFR [human]-H10. Thioredoxin was not as effective a solubility enhancing tag when fused at the C-terminus with an average soluble yield of only 0.7 mg/l compared with 6.0 mg/l when fused to the N-terminus."

We are aware that other studies (e.g. referenced in the Discussion of the BMC Biotechnol. paper) came up with partly different rankings. On the other hand, in our own previous work (Journal of Virology, 2012, 86: 10079-10092), we found that C-terminal MBP fusion efficiently increased the solubility of truncated duck hepatitis B virus polymerase which has a notoriously low solubility in *E. coli*.

Altogether, there seems to be no full consent in the literature. This likely reflects the multifactorial nature of "soluble expression" which is affected by many more parameters than just the tag and its position. Such factors include, besides temperature, the translation vs. folding rate (slower translation promotes more efficient folding), the absolute concentration of the recombinant protein (lower concentrations suppress aggregation), and these will again depend on factors such as promoter strength, ribosome binding site, and start codon accessibility (which depends on the actual nucleotide sequence around the AUG). In the end, each specific fusion protein may display a set of individual properties, as summarized in a recent methods paper (Steinmetz and Auldridge. 2017. Screening Fusion Tags for Improved Recombinant Protein Expression in E. coli with the Expresso® Solubility and Expression Screening System. Curr Protoc Protein Sci. 90: 5.27.1-5.27.20): "Although several different fusion partners have gained favor, none are universally effective, and identifying the one that best improves soluble expression of a given target protein is an empirical process."

Given these conditions we agree that our previous conclusion from Fig. 3, namely that the "P17 tag was better than MBP to increase scFv's solubility in SHuffle T7 cells", was too general and needs to be qualified in terms of position, namely that "A C-terminal P17 tag enhanced G12-scFv solubility more efficiently than C-terminally fused MBP in SHuffle T7 cells". In line with the citation above we also examined another commonly used tag, glutathione-S-transferase (GST), reported to be more efficient when N-terminally fused to the passenger protein (Malhotra, Meth. Enzymol., 2009, 463: 239-258). However, the proportion of soluble to total GST-G12-scFv protein was marginal (modified Fig. 3). Hence the 33-aa P17 tag at the C-terminus was much more efficient for enhancing scFv solubility than N-terminal GST and also more efficient than C-terminally fused MBP whose length of >350 aa puts a much larger metabolic burden on the producer cell.

**Minor Points:**

1.- Line 81: The authors state that the rapid clearance of scFv is advantageous, but I have read many times that it's the opposite. That IgG is retained longer in the body and has longer lasting effect. Authors should clarify that in certain conditions, rapid clearing could be preferred but is not necessarily an advantage.

R1: Thanks for the suggestion! We fully agree that the pros and cons of rapid clearance depend on the application. We have clarified the specific conditions under which the better tissue penetration and more rapid clearance of an antibody fragment compared to a complete antibody are advantageous, by the following sentence starting from Line 77 of the current version: "Due to their smaller sizes, Ab fragments have higher tissue penetration capability and are more rapidly cleared than mAbs, making them particularly suitable for applications such as diagnostic in vivo imaging and therapeutic targeting of tumors and intracellular infectious agents including viruses". The phrase "Despite these advantages" in Line 84 of the former version was also changed to "Despite these small-size related benefits" in Line 84 of the revised version.

2.- Line 107, 113, 132 and 174: Several times in the manuscript, gene names such trxB and gor along with in vitro and in vivo were not italicized. Please go over the manuscript and correct.

R2: Thanks for the hint. Gene names and the phrases “*in vitro*” and “*in vivo*” were all italicized in the current version of the manuscript.

3.- Line 109: Isn't DsbA a thiol disulfide-bond oxidase instead of a sulfhydryl oxidase?
R3: We agree that "sulfhydryl oxidase" usually refers to eukaryotic enzymes that oxidize 2 thiols to a disulfide by reducing $O_2$ to $H_2O_2$ while DsbA, as defined by the Integrated relational Enzyme database (IntEnz), "contains a redox-active disulfide bond that is catalytically transferred via disulfide exchange to a diverse range of newly translocated protein substrates. The protein is restored to the oxidized state by EC 1.8.5.9, protein dithiol:quinone oxidoreductase DsbB." The accepted names of DsbA are thiol-disulfide oxidoreductase or, systematically for E.C 1.8.4.15, protein dithiol:[DsbA protein] oxidoreductase (protein disulfide-forming). Then we now use thiol-disulfide oxidoreductase throughout the text.

4.- Line 130: “…T7 phage tail can target…X,Y,Z…” to what?
R4: The answer is: to hepatocytes. The complete sentence from Line 130 to Line 132 of the former version reads “The P17 protein of T7 phage tail can target phage particles and other cargos including small molecules, proteins, siRNA, DNA polyplexes, and liposomes to hepatocytes in vitro and in vivo.”

5.- Line 140: What is a “sequential” property? Do the authors mean the property of the peptide coming from the sequence? What is the difference between the sequence of a peptide and the structure it forms? Please clarify
R5: Sorry for the ambiguous statement in Line 140 of the former version. The meaning of "sequential" was not temporal but "sequence-related".

Regarding the difference between sequence and structure of a peptide: while sequence generally determines structure not all parts of a peptide or protein need to be structured. For instance, interactions with other molecules may occur through an unstructured loop yet binding may well be affected by the specific aa sequence of that loop. Conversely, a specific structure, e.g. an a-helix or parts of it (see reviewer #2), may act as an interaction interface yet a specific aa presented within that structure may allow or prevent that specific interaction. Further examples are antibodies themselves, some of which recognize linear and others conformational epitopes.

We modified the previous sentence “Therefore, sequential and structural properties of P17 peptide for the solubility-enhancing ability were extensively investigated in the current study” to now: “The current study aimed to identify the sequence and structure determinants within the P17 peptide that are responsible for its solubility and stability enhancing impact on scFvs, and to clarify the underlying mechanisms.” in Lines 145-147 of the new version.

6.- Line 170: Are the two decimal points significant and reproducible? If not please round up.
R6: We agree with the reviewer. While the solubility differences were fully reproducible and significant (see Fig.3) the densitometric assay as such does not allow for double-decimal resolution. Following the reviewer’s suggestion solubility percentages were rounded up to one decimal point, here 14.9% for G12-scFv-HA and 45.0% for G12-scFv-HA-P17. See also point 18 of this reviewer.

7.- Line 195: Authors claim that HA tag did not contribute to folding. But if one compares in Fig 2b the lanes 4 to 6, one can clearly see a negative effect of having HA. Please correct that HA does have an effect.

R7: Thanks for pointing out this mistake. As shown in Fig. 1d and the new Fig. 2d, attachment of an additional HA tag indeed reduced solubility enhancement by the P17 tag. We corrected our description by adding two sentences in Line 201 of the current version "… indicating that the additional presence of the HA tag decreased solubility enhancement by the P17 tag." and in Line 211 "Therefore, direct attachment of the P17 tag to either terminus of the scFv increased solubility efficiently, whereas an additional HA tag at the scFv C terminus reduced the P17-mediated solubility enhancement."

8.- Line 219: The description "P17 tag may be acquired" is confusing. Please clarify and re-word. Perhaps, "may be attributed to…"

R8: For clarification, the sentence in Line 219 of the former version "Solubility-enhancing ability of P17 tag may be acquired by recruiting other molecular chaperones to promote correct protein folding." has been modified to: "The solubility-enhancing ability of the P17 tag may be endowed by recruiting other molecules such as chaperones or perhaps tRNAs to promote protein folding." in Line 233 of the revised version.

9.- Line 236: The authors claim to disrupt the helix by inserting a proline but have not demonstrated that the proline substitution had any effect on the folding of P17. Please soften the conclusion to indicate that the proline substitution did not have any observable effect, potentially suggesting that the exact nature of the helix does not play a significant role, according to this single mutant construct.

R9: Thanks for the suggestion. As α-helix formation was also a major issue for reviewer #2 we re-summarize here the arguments and data favoring and disfavoring the existence or formation of such an α-helix and its most likely localization within the P17 tag.

1) There are no direct structural data for the 533 aa full-length gp17 available that cover the P17 tag sequence; the sequences of the published structures end before (e.g. PDB 7BOZ) or start after the P17 tag sequence (e.g. PDB 4A0T).

2) By circular dichroism studies done by Wong et al. (Molecular Pharmaceutics, 2006, 3: 386-397) found no evidence for the existence of an α-helix in the P17 peptide.

3) Predictions:

- in an early study on the full-length 533 aa gp17 tail fiber protein (J. Mol. Biol., 1988, 200: 351-365) the authors noted a high α-helix propensity by the 1978 algorithms of Chou/Fasman and Garnier in the "rod-region" (aa 151-267) which contains the P17 sequence (aa 186-218); this has not been experimentally confirmed yet (see 1).

- newer predictions including Jpred4 (Nucleic Acids Res., 2015, 43: W389-94) suggest indeed an α-helix propensity for the P17 peptide sequence $R_{22}$ - $K_{29}$ in the P17 tag (corresponding to $R_{207}DEA_{210}KRFK_{214}$ in full-length gp17 protein), as shown in the new Supplementary Figure 1a. Due to the helix-breaking property of Pro the α-helix score drops drastically when the central A25 is replaced by P (Fig. S1a). By contrast, mutations $D_{23}E_{24}$>AA, $K_{26}R_{27}$>AA and their combination maintain the α-helix propensity. Hence if an α-helix formed in the wild-type

(wt) P17 tag it should be preserved in these mutants, which then would test for the loss of charges in α-helical context (see also reviewer #2) - or if no α-helix forms anyway just for the impact of loss of charges.

- In addition, we used AlphaFold2, one of the most advanced protein structure prediction tools, to model protein MA18/7-scFv-HA-P17 carrying the wt P17 tag or its A25P mutant. The predicted local Distance Difference Test scores (plDDTs; from 0-100, with >90 regarded as indicating very high confidence and 70-90 good confidence in the predictions) were low at the N terminus, in the center of the chain (~position 140 at the Gly-Ser linker for VL-VH junction) and near the C terminus of the scFv sequence; however, towards the very end, representing the P17 tag, a plDDT score of at most 60 in the best of five models for the formation of an α-helix in the C terminal part of the P17 tag was predicted. In line with a low helix propensity, plDDTs for the next best models dropped to below 40 (with <50 often regarded as disordered). For the A25P mutant, the scores for the entire P17 tag remained below 40, in line with a very low α-helix propensity, in AlphaFold usually presented as a long filament (J. Mol. Biol., 2022, 434: 167336). Still, both the wt P17 tag as well as its A25P derivative fused to the C terminus of MA18/7-scFv-HA similarly increased solubility by around 4-fold (Fig.5b, 5c, and 5d). Moreover, deletion of the potential helix forming sequence (variant P17-C15del, Fig. 6) still exhibited some solubility enhancement, though to a reduced extent.

Notably, reviewer #2 suggested in his major point 4 (SEE BELOW) the possibility that such an α-helix, with an uneven distribution of positively charged side-chains might recruit tRNA which could then act as chaperone ("Chaperna", see below). In this context the additional mutants in the putative α-helix region are of interest, wherein two acidic or two basic residues, or all four charged residues together were replaced by alanine (Fig. 7); replacements of the DE dipeptide and the four charged aa DEKR had the strongest negative impact on solubility enhancement, while replacement of the KR motif had not much effect.

Rather than this long textual description we have added a new supplementary figure (Fig. S1) which summarizes the pertinent prediction data for α-helix formation within the P17 tag.

Altogether, our data do not support a functional importance of a putative α-helix in the P17 tag. However, following the reviewer´s suggestion we still tuned down our conclusion on the lacking importance of the putative α-helix by stating: “the putative α-helix structure, if formed at all, was dispensable for solubility-enhancement” (see Line 267 of the revised version).

10.- Line 249: Why did it authors truncate by 8 and 15 aa? Please explain

R10: The aim of deleting 8 and 15 aa from C terminus of P17 tag was to partly and completely remove the 8-aa sequence predicted to possibly form an α-helix (see R9 and the new Supplementary Figure 1). We reworded the sentence in Line 278 of the current version as follows: “P17 mutants P17-C8del and P17-C15del lack 8 and 15 aa from the C-terminus to partly and completely delete the sequence forming the predicted α-helix.”.

11.- Line 249: Who is “they”?

R11: They refer to P17-C8del and P17-C15del mutants of P17 tag. To clarify this, we reworded the sentence beginning from Line 278 of the current version: “P17 mutants P17-C8del and P17-C15del lack 8 and 15 aa from the C-terminus and thus partly and completely delete the putative α-helix sequence. Attached to the C terminus of MA18/7-scFv both truncated tags still improved solubility significantly by 7.3 and 5.9 fold, respectively (Fig. 6b, 6c, and 6d)...”.

12.- Line 301: The authors claim that P17 stablizes scFv by “inhibiting exposure to hydrophobic regions” but at this stage this is very speculitave and they finish the paragraph by stating that P17 promotes protein folding. Please clarify, whether its protein folding or inhibiting exposure to hydrophobic regions or both and what the evidence for these claims are and re-word.

R12: Please see also R3 to reviewer #2, minor point. We agree that mechanistic interpretations remain speculative at this stage. However, as also discussed below, the term "promotes folding" in a wider sense includes the prevention of non-productive interactions - which indirectly facilitates productive interactions (leading to the proper 3D fold). Many researchers would probably agree that this is an important aspect of how general chaperones "promote folding" of thousands of different clients. Even ATP-dependent foldase processes may be viewed from this angle, except that they actively provide additional chances for trapped improper folding products "to try again".

Our experimental approach to compare stabilities of different scFv derivatives indeed measured thermally induced <u>exposure of hydrophobic regions</u> by the increased binding of a hydrophobic fluorescent dye (SYPRO Orange). Hence we consider our conclusion in Line 330 "…indicating that P17 tag stabilized scFv by inhibiting exposure of hydrophobic regions" was properly phrased.

At the end of the paragraph beginning from Line 293 of the former version, we stated “However, incubation with in vitro synthesized P17 peptide even in 10-fold excess failed to increase Tm of G12-scFv-HA (Fig. 8 bottom panel), indicating that P17 tag stabilized scFv only as part of the protein, possibly by promoting protein folding.” We considered the wording of the last half-sentence as sufficiently cautious to underline its speculative character. However, as reviewer #2 also raised this point we followed his suggestion and rephrased to (from Line 331 of the current version): “As a control, incubation in trans with in vitro synthesized P17 peptide failed to increase the Tm of G12-scFv-HA even in 10-fold excess (Fig. 8 bottom panel), indicating that the P17 tag stabilized scFv only as part of the protein and did so <u>by increasing resistance against unfolding.</u>”.

13.- Line 311: which small surface protein?

R13: Thanks - we added “HBV” to define the source of small surface protein; the same in the legend to Fig. 9a.

14.- Line 325: should be “self-assembly into dimers”

R14: Thanks - has been corrected.

15.- Line 331: aren’t the ranges from 0.137 kD to 670 kD? There are two peaks, one at 13.7 and the other 0.137.

R15: Yes, protein markers range from 0.137 kDa to 670 kDa. Thanks for pointing this out.

16.- Line 404: the authors claim that there is no need to remove the P17 tag, but do not show any evidence of this. Does the cargo remain soluble after the removal of P17? If as the authors claim, P17 inhibits exposure to hydrophobic regions, will it remain to do so after the folding of scFv? Also, the authors state that “there is no need to cleave off the P17 tag” but I argue that choice is upto the user and the needs of the experiment. The authors should clarify that the user has the choice to retain the tag, should they want to and that scFv-P17 fusion can still be used for binding studies, should they want to.
R16: We agree with the critiques of reviewers #1 and #2 about our initial statement that there is "no need to remove the P17 tag from the scFv"; of course, this depends on the intended application. For some applications the tag may stay on but for others removing the tag is probably crucial - but only if this does not induce re-exposure of hydrophobic scFv regions, insolubility and aggregation.

We addressed these issues by new experiments placing an enterokinase recognition site (DDDDK) between the C terminus of scFv and the P17 tag, giving protein G12-scFv-ETK-P17 (Fig. S3c). Only 0.012 units enterokinase (Yeasen, Shanghai; one unit is defined as the amount of enzyme that will cleave 500 µg of fusion protein in 12-16 hours at 25°C, in a buffer containing 25 mM Tris-HCl, pH 8.0) were sufficient to completely clip-off the P17 tag from 22 µg of the fusion protein. Importantly, centrifugation of the digestion reaction at 20,000g for 30 min did not yield any visible pellet; more sensitively, Coomassie-Blue staining band intensities from the same volumes of untreated and enterokinase-treated fusion protein after centrifugation remained virtually the same, indicating that no loss of soluble protein had occurred during protease treatment. In addition, no undesired cleavage products were formed.

We then modified our statement starting from Line 439 of the current version as: “Therefore, the P17 tag removal is not mandatory for *in vitro* use. However, if desired, especially for *in vivo* applications, the P17 tag can be efficiently cleaved off by enterokinase, and possibly other cleavage site/protease combinations, to avoid the potential immunogenicity (Fig. S3c) after attached to scFv via an enterokinase digestion site (DDDDK). Importantly, solubility of the tag-less scFv protein was not compromised, as shown by virtually identical band intensities of equal amounts of untreated vs. enterokinase-treated protein after centrifugation at 20,000 g for 30 min. These data also suggest that the P17 tag facilitates the adoption of a stably folded scFv conformation but is not required to maintain it.”.

17.- Line 458: “P17 tag could also may be excluded”
R17: “P17 tag” has been removed in the new version.

18.- Figure 1b/c: Are the two decimal points in % solubility significant? If not, round up.
R18: We rounded up the percentage values of solubility in Fig. 1b/c and other figures to one decimal point.

19.- Figure 1d: The constructs should be in order of solubility

R19: Constructs in Fig. 1d are now shown in the order of fold changes of solubility of P17 tagged scFv vs. untagged scFv.

20.- Figure 5: More features of the P17 tag should be shown, such as residue numbers, charged residues, kD and pI of the tag and other secondary structures such as helices and strands if any and the mutant proline should be red as in Fig 7A. It might also be worth showing the predicted structure of the tag from Alphafold and or from a real structure, if any.
R20: Thanks for the suggestion. Residue numbers of the P17 tag sequence are now labeled in Fig. 5-7. Positively and negatively charged as well as polar residues of the P17 tag are shown in blue, red, and green in Fig. 7, respectively. Isoelectric points (pIs) of various scFvs are listed in the table of Fig. 1d. Proline (P) 25 of P17-A25P mutant is shown in red in Fig. 5a. See also the new Supplementary Figure 1.

Currently no real structures of the full-length P17 protein or the free P17 tag are available (see above); the protein database (PDB) has several entries with high resolution structures of the C terminal P17 sequence, e.g. 4A0U with residues 371-553, and of the N-terminal part, e.g. 7BOZ covering the structure of residues 1-144 but none covering the P17 tag part (aa 186-218). However, we have now indicated, as suggested, the aa sequence of residues 22 to 29 predicted to fold into an α-helix as a red cylinder (as from Jpred4) in Figs. 5-7. In addition, we show the AlphaFold predicted models of MA18/7-scFv-HA-P17 protein and its P17-A25P variant containing a C terminal P17 tag and a P17-A25P mutant respectively in Supplementary Figure 1b.

21.- Finally, looking at the first and last residues of the P17-tag, is it possible for the authors to think of NEST-TAG instead of P17 tag? :)
R21: Thank you for this interesting suggestion. However, others have recently published a preprint on a sequence-wise unrelated 53 aa "NEXT-TAG" (bioRxiv, 2021, https://doi.org/10.1101/2021.08.05.455358), derived from carbonic anhydrase of the marine bacterium *Hydrogenovibrio marinus*. Such similarity in names would be prone to cause confusion with a "NEST-TAG". In addition, our data show that the very terminal residues of the P17 tag are largely dispensable for solubility enhancement; hence will abstain from this principally appealing suggestion.

**Reviewer #2**

While the paper interesting in exploiting intramolecular chaperone function for the folding of svFv, the main conclusions are based on only preliminary data, and requires further extensive analyses as suggested below.
R: As outlined below we do not agree that our conclusions are "based on only preliminary data"; however, we also appreciate several inspiring comments which prompted us to perform additional experiments.

**Major Points:**

1. A major criticism on the claim for promoting the solubility. The soluble fraction was defined throughout the text as the fraction that remained soluble after addition of SDS buffer to the ‘clear supernatant’ (M&M p21, line 621). Traditionally, the ‘clear supernatant’ (in this paper) has been defined as ‘soluble’ fraction in most of prior arts to address to their abilities to enhance their solubility and thus the productivity of the protein of interest.

R1: We assume there is a misunderstanding about our definition of "soluble". We fully agree with the reviewer that solubility "AFTER ADDITION of SDS buffer" would not allow for any conclusions on a solubility-enhancing effect of the P17 tag (or any other tag), and hence would make our claims on solubility enhancement by the P17 tag obsolete. However, in agreement with the reviewer´s view we had defined the "clear supernatant of the bacterial lysates after centrifugation" as the soluble fraction. Only AFTER separation into the soluble and insoluble fraction of the lysates by centrifugation was SDS sample buffer added for the subsequent SDS-PAGE analysis of the two fractions. Hence our solubility data are fully in line with the "traditional definition", and we consider our conclusions as valid. However, to exclude any further misunderstandings we have modified the text as follows: “After centrifugation of bacterial lysate at 12,000g at 4°C for 20 min, 10 μl clear supernatants representing soluble fraction were mixed with 40 μl 2×SDS-PAGE loading buffer for Western blotting analysis of soluble scFv protein” beginning from Line 653 of the current version. Accordingly, the words to describe “total bacterial protein fraction” were also modified as follows: “Ten μl bacterial lysate representing total bacterial protein fraction was mixed with 40 μl 2×SDS-PAGE loading buffer (100 mM Tris-HCl, pH 6.8, 4% SDS, 12% glycerol, 4 mM DTT and 0.02% bromophenol blue) for Western blotting analysis of total scFv protein” starting from Line 649 of the current version.

2. Because of a rather narrow definition of solubility adopted in this m/s, it is really impossible to calculate the overall yield of the scFv proteins. In fact, the overall yield was not mentioned throughout the text, which should be an integral part for adopting the microbial production with a view to address to the cost and yield (Intro. p3, line 84; Discussion. P12, line 351).

R2: We agree that the overall yield is an integral part for considering the potential benefits of microbial scFv production. Even if the P17 tag could mediate production of 90% of the scFv in soluble form this would make little sense if the overall yield per liter culture was in the microgram range. However, we respectfully disagree with the reviewer´s conception that "the overall yield was not mentioned throughout the text". We actually determined overall soluble yields of G12-scFv and G12-scFv-P17 (see Fig. S3b) to be 3 mg and 8 mg per liter culture, as already mentioned in the text from Line 293 to 296 of the former version. Therefore, the P17 tag increased the yield of soluble G12-scFv by ~2.7 fold, in line with solubility enhancement by the P17 tag. In addition, in the revised manuscript we also determined the soluble yield of G12-scFv-ETK-P17 with a enterokinase digestion site between G12-scFv and P17 tag to be 6.3 mg per liter culture (Fig. S3b), further corroborating the solubility enhancing effect of the P17 tag.

3. The data is lacking on the removal of the P17 tag after purification. The cleavage and the final yield is an essential part of the paper, especially because of potential immunogenicity of the p17 tag in the fusion construct. The authors claim - “Therefore, there is no need to cleave off the P17 tag after purification of scFv-P17 fusion protein” (Discussion. P14, line 405)-

cannot be justified from the view of regulatory process. Otherwise, the authors should provide an evidence that the P17 tag is not immunogenic.
R3: We thank the reviewer for this constructive comment, also raised by reviewer #1 as minor point 16 (see above). We agree that for various applications, particularly in vivo, the presence of heterologous sequences on an antibody/antibody fragment can be problematic; a large tag like MBP could occlude the antigen binding site and, while this is unlikely for a tag as small as P17, either tag may have immunogenic potential on its own, an undesired property especially in therapeutic applications. As suggested, complete removal of the tag would be the safest option - unless the scFv protein would aggregate after tag removal.

To address this point we generated additional constructs carrying an enterokinase recognition site between scFv and the C terminal P17 tag. Treatment with enterokinase resulted in efficient removal of the P17 tag without causing insolubility/aggregation of the released scFv; these new data have been provided as the Fig. S3c in the revised manuscript. In addition, we rephrased the statement that P17 tag removal would not be necessary to make a clear distinction that this is a question of the intended application: see also minor point 16 by reviewer #1 for details.

4. A major debate arises on the mode of action of the P17 tag as an intramolecular chaperon. Specifically, the conclusion that the action is independent of the proposed α-helix secondary structure (Abst. 02, line 43) is based on a limited mutagenesis on the sequence of P17 (as shown in Fig 6 & 7), and a skewed view that chaperone is proprietary of proteins. The present reviewer executed the heptad repeat helical wheel depiction of P17, which reveals a 'sidedness' of positive charge of the alpha-helix, which can interact with negatively charged molecule, such as tRNAs of high molecular abundance inside the bacterial cytoplasm. (Kwon et al, Int J Mol Sci. 2018 Oct 3;19(10):3016) providing chaperone function. It merits further analyses on potential transition of P17 tag from the disorder into alpha-helix induced by tRNA interaction, and whether the proposed intramolecular chaperone function of P17 tag (Abst. P2, line 41) is actually mediated by tRNA interaction (refer to recent articles on Chaperna (RNA as chaperones) on the folding of client proteins; Nanomedicine. 2021 Oct;37:102438.; Biomaterials. 2021 Feb;269:120650; Biotechnol Bioeng. 2019 Mar;116(3):490-502). More specifically, further mutations on positively charged residues (single, or multiple mutations) in Fig 6 should be analyzed before suggesting the mechanism of the chaperone function; whether chaperone function is mediated by tag alone (as suggested by the authors) or by interaction with RNA molecules. Does the fusion protein co-purify with RNA molecules? (refer to Biomaterials. 2021 Feb;269:120650).
R4: The term *internal chaperone* was originally coined for naturally occurring intra-protein sequences which help the client protein to adopt a specific 3D (type I) or quaternary structure (type II), and then are often clipped-off (Curr. Opin. Struct. Biol., 2008, 18: 765-770). The term is also used more broadly to describe a solubility- or folding-promoting activity of several "protein-tags" in artificial fusion proteins; this is the meaning we here use. We agree that our analysis of the P17-mediated solubility enhancement does not provide a full mechanism-of-action; notably such data are hard to find for other tags as well. However, we considered it relevant to define which parts or features of the P17 tag are responsible or dispensable for the solubility enhancing effects on scFvs, in particular whether a putative α-helix is a potentially important feature.

Here we appreciate the reviewer´s reference to recent publications (Int. J. Mol. Sci., 2018, 19: 3016; Nanomedicine, 2021, 37: 102438; Biomaterials, 2021, 269: 120650; Biotechnol. Bioeng., 2019, 116: 490-502) on a novel RNA-based chaperoning mechanism ("chaperna"). Prototypic is the disordered RNA interaction domain (RID) of eukaryotic lysyl-tRNA synthetases whose basic aa residues mediate the interaction with tRNA, an abundant component in the bacterial cytoplasm. This interaction can induce α-helix formation in the RID, with the RNA taking a chaperone role ("chaperna"). Meanwhile, RIDs have been engineered into artificial RNA-dependently assembling ordered multimers, including virus-like particles. As suggested by the reviewer, tRNA binding to helical regions of P17 tag and/or α-helix induction by tRNA might provide a mechanism for P17-mediated solubility enhancement.

While our previous data made it already unlikely that α-helix formation by P17 is crucial for solubility enhancement (which was largely maintained when the central A residue in the putative α-helix (residues $R_{22}DEA_{25}KRFK_{29}$) was mutated to α helix-breaking P) we performed additional experiments to evaluate a potential role of RNA for P17 tag function.

Regarding the likelihood for α-helix formation within the P17 tag see our response to minor point 9 of reviewer #1 and the new additional Supplementary Figure 1.

First we tested, as suggested by the reviewer, whether tRNA would co-purify with the P17-tagged fusion proteins - which it did not, as shown by the electrophoretic mobility shift assay (EMSA) in the new Supplementary Figure 2. Furthermore, in the cited paper (Int. J. Mol. Sci., 2018, 19: 3016) Kwon et al. found that the 8.3 kDa RID showed a strongly retarded electrophoretic mobility in SDS-PAGE (appearing like a 20 kDa protein) due to tRNA binding; in contrast, our P17-tagged vs. P17-lacking fusion protein migrated during SDS-PAGE at the expected ~ 30 kDa position.

Moreover, as requested we performed additional mutagenesis studies targeting the charged dipeptides $D_{23}E_{24}$ and $K_{26}R_{27}$ in the putative α-helix, replacing them individually and in combination by 2 A residues each. Then we assessed their impact on solubility enhancement of the MA18/7-scFV-HA protein in the context of the shortened P17 variant N18del which maintains aa 19-33 comprising the putative α-helix residues 22-29. Replacement of the KR motif had little negative effect (~12%), that of the DE motif more (~42%), and replacement of both motifs reduced solubility enhancement by nearly 60% compared to the unmodified N18del peptide. Hence the charged residues do play a relevant role in solubility enhancement by the P17 tag, but there is no evidence that this involves α-helix formation and/or tRNA binding as the actual chaperoning activity of the P17 tag.

As these data may be of interest for a wider readership we have included the most pertinent results into the new Supplementary Figure 2 and the main text (starting from Line 254) as follows: “However, the α-helix might still form in the scFv context or be induced by interaction with bacterial components, notably the abundant tRNA, as in the recently described "chaperna" mechanism where RNA is crucial for chaperoning. Different from the RNA interaction domain

used there, we could not detect a stable association of the P17 tag with RNA (Fig. S2), disfavoring such a mechanism." .

**Minor points:**

1. Describe how was the signal quantified. Densitometric scanning of the gel? (p6 lines 159-161; p7 line 185)

R1: We employed densitometric scanning of the Western blotting signals, recorded by a charge-coupled device camera, using Multigauge V2.2 software (Fujifilm) for semi-quantitative determination of the relative band intensities in the total and soluble fractions of scFvs. To make this clearer to readers we added a few explanatory words in Line 164 of the revised version: "…both fractions of scFvs were semi-quantitated by Western blotting using a mAb against the common His tag followed by a horse-raddish peroxidase conjugated secondary antibody plus a chemiluminescent substrate, and subsequent densitometry of the blotting signals recorded by a charge-coupled device camera (Fig. 1a upper panel)."

To account for the limited dynamic range of the method, we used different dilutions to avoid signal saturation. All experiments were performed at least three times.

2. "Direct or indirect fusion" (p7, line 203)? The meaning of 'indirect fusion' is unclear.

R2: Indirect fusion was meant to indicate that the P17 tag was not directly attached to the C terminus of scFv but rather "indirectly" via an already present HA tag (Fig. 1c and 1d). Since this HA tag reduced solubility enhancement by P17, we performed additional experiments to compare the effect of N- vs. C-terminal location of the P17 tag on the solubility of MA18/7-scFv containing, or not containing, the HA tag. Hence, the respective results were reworded (in Lines 204 to 213 of the revised version, also see the detailed response to minor point 7 of this reviewer).

3. "Promoting protein folding" (p11, line 304)? May discuss on the possibility of increasing the stability of the folded protein & resisting the protein unfolding.

R3: Please refer to our detailed response to minor point 12 of reviewer #1 who raised the same question. "Promoting folding" is an often somewhat loosely used term to describe an enhancement of the final fraction of folded vs. unfolded or misfolded protein, regardless of the underlying mechanism(s); in fact it seems that despite many efforts, rock-solid mechanistic explanations are rare. A major mechanism employed by professional chaperones is the prevention of unproductive interactions between still unfolded or not yet finally folded, mostly hydrophobic parts of a client protein; these would otherwise (especially at high concentration) lead to insoluble aggregates. In this way chaperones can "assist folding" of many diverse clients, including by the active (ATP-consuming) resolution of non-productive folding intermediates. The situation seems different for intrinsically disordered proteins or protein domains where a specific interaction partner may act as template/mold for the disordered domain to adopt a specific structure (see also above, RID-tRNA interaction).

What our thermal shift assays actually show is a 2.3°C higher Tm for the P17-tagged G12-scFv-HA vs. the untagged version, demonstrating that the tag indeed increased the fusion protein´s resistance/resilience against unfolding. Following the reviewers´ suggestion we have reworded

the corresponding sentence from "by promoting folding" to "by increasing resistance against unfolding" in Line 334 of the new version.

4. 2-fold difference in binding affinity (p11, lines 313-314): Is it significant?
R4: We agree that the 2.2-fold difference in binding affinity appears not so obvious. We therefore performed additional ELISA experiments which corroborate that the 2.2-fold difference in binding affinity is indeed significant (Lines 344 to 350 of the revised version).

For the respective ELISA assays microtiter wells were coated with 5 µg/ml P17-tagged or untagged G12-scFv, and then reacted with pooled sera of chronic hepatitis B patients. The absorbances at 450 nm (A450) for P17 tagged G12-scFv was significantly ($p<0.0001$) ~2 fold higher than that of untagged version (Fig. 9b); these data further corroborate a higher antigen-binding affinity of the P17 tagged protein, probably via a higher proportion of properly folded G12-scFv.

5. "Type 1 vs type 2 intramolecular chaperone" (p16, lines 465-467). The distinction is not rigorous in the area of protein folding and chaperone. Usually, the same chaperone provides the quality control of the client proteins by assisting folding and assembly cooperatively (Science. 2017 Dec 8;358(6368):1272-1278; Biomaterials. 2021 Feb;269:120650).
R5: The term intramolecular chaperone initially referred to a terminal extra sequence in a native protein that is required for the folding of that protein, or for its assembly into higher order quaternary structure; often the extra sequences are removed after their job is done (reviewed in Chen and Inouye. The intramolecular chaperone-mediated protein folding. Current Opinion in Structural Biology, 2008, 18: 765-770). By their definition, intramolecular chaperones of type I assist folding into tertiary structure, those of type II quaternary assembly. The elegant studies referred to by the referee show that chloroplast chaperones (Science, 2017) and tRNA (Biomaterials, 2021) can efficiently assist in the assembly of multi-subunit complexes, i.e. Rubisco and virus-like particles, but they do not specifically refer to intramolecular chaperoning.

In our experiments the P17 tag enhanced solubility of different scFvs expressed in *E. coli* SHuffle cells irrespective of its N- or C-terminal localization. The P17 tag was monomeric on its own and we found no evidence that it promoted higher order quaternary structure of scFv (Fig. 10). Moreover, post purification provision of excess P17 peptide *in trans* did not increase thermostability of G12-scFv (Fig. 8 bottom panel). These properties are consistent with the classical definition of an intramolecular type I chaperone, even if the type I vs. type II distinction is not rigorous in other cases. We would therefore prefer to maintain this nomenclature in our study.

6. "non-ATP hydrolyzing chaperones" (p17, lines 482-483). Better described as foldase (ATP-dependent) and holdase (ATP-independent) type chaperones (Nat Commun. 2021 Feb 8;12(1):851).
R6: We thank the reviewer for this suggestion. We then modified the respective wording from "non-ATP hydrolyzing chaperones" to "ATP-independent holdase chaperones" and cited the paper mentioned by the reviewer in Line 522 of the revised manuscript.

7. Fig 2b. With respect to comparing C-terminal vs N-terminal fusion, additional data required comparing MA18/7-P17 and P17-MA18/7.

R7: As per the reviewer's request, we compared the solubility of MA18/7-scFv with the P17 tag attached to either end in the absence of the additional HA tag. As shown in Fig. 2b and Fig. 2c, the P17 tag significantly improved solubility at both N- and C-positions by 11.6 and 10.2 fold, respectively. When fused to a C terminal HA tag the P17 tag increased solubility by 5.9-fold while at N terminus the improvement was 3.5-fold (Fig. 2d). Therefore, direct attachment of the P17 tag to either terminus of the scFv (see also Minor point 2) increased solubility efficiently, while the additional presence of a C terminal HA tag reduced solubility enhancement by the P17 tag. The related description of the results was revised accordingly (Lines 204 to 213 of the current version).

8. Fig 5. The mutation Ala into Pro is at the distal end of P17, and is not expected to influence the helix formation strongly. Mutation on the positively charged aa in heptad repeat may better address to the mechanism of chaperone action (Major point 4).

R8: We have addressed the issue of a-helix formation in P17 tag in detail in our responses to point 9 of reviewer #1 and major point 4 of this reviewer. In brief, there is no experimental evidence for helix formation in P17 tag, and up-to-date prediction algorithms point to only one region with a propensity for α-helix formation, comprising the sequence $R_{22}DEA_{25}KRFK_{29}$ (new Supplementary Figure 1). Pro is incompatible with the typical features of an a-helix, hence Pro is generally considered as helix-breaker. Hence first, while at the distal end of P17, the A25P mutation is right in the middle of the only region with a reasonable helix propensity (new Supplementary Figure 1); hence we respectfully disagree with the reviewer´s view that a "Pro at this position is not expected to influence the helix formation strongly".

Rather, the up-to-date predictions by Jpred4 and AlphaFold2 strongly support the view that IF an α-helix would form in the wt P17 tag, it would be destroyed by the A25P mutation (new Supplementary Figure 1); this is further supported by the AlphaFold2 models for the MA18/7-scFv with wt P17 tag vs. the A25P variant (new Supplementary Figure 1).

Importantly for interpretation, the A25P mutation had no substantial impact on solubility-enhancement by the P17 tag (Fig. 5), suggesting the putative α-helix is not crucial for this function. We also examined additional mutants, in particular of positively as well as negatively charged residues. Double Ala substitution of K26R27 had only a slight and that of D23E24 a larger negative impact on solubility enhancement; the worst case was replacement of all four charged residues by the uncharged A residues (Fig. 7). As outlined above, we therefore conclude that the charged and polar residues in P17 are most important for solubility enhancement by the P17 tag but without a requirement for their presentation in an α-helical context.

**Reviewer #3:**

The authors reported that a 33-residue peptide named P17 tag potently increased solubility of multiple scFvs produced in E. coli SHuffle strain, especially if attached at the carboxyl terminus. Solubility-enhancing effect of P17 tag was mainly attributed to its hydrophilic

sequence especially ionic residues, but independent of the proposed α-helix secondary structure. Remarkably, P17 tag elevated thermostability of scFv as efficiently as intra-domain disulfide bonds. Moreover, P17-tagged G12-scFv targeting surface protein of hepatitis B virus showed over two-fold higher antigen-binding affinity and virus-neutralizing activity than untagged version. These results is interesting and have potential significance to the related readers. Other comments were as followed.
R: We thank the reviewer for the positive general comments and acknowledging that our results are interesting and have potential significance to the related readers - but, as we infer from the questions and comments, they may be considered as being of limited novelty. Below are our responses to the reviewer's questions and comments, with emphasis on the novelty aspect of our data.

1. There are many scFv publications focused on scFv cloning, expression, purification, characterization, especially on solubility. So the novelty of this article is not higher enough.
R1: We respectfully disagree with this comment. The many publications referred to by the reviewer reflect the still ongoing quest for improving the production of scFvs - as "minimized mAb mimics". This minimizing has principally enabled prokaryotic expression of mAb-like proteins. However, major challenges remain to generate them in sufficient quality and quantity, and at reasonable cost (a major driving force for using prokaryotic hosts) to be useful for diagnostic and therapeutic applications. Two of the most important yet persistent challenges are **low solubility and stability** (Frontiers in Microbiology, 2020, 11: 1927), which are in the focus of our study (rather than cloning, purification, and characterization of scFvs). The main finding was the solubility-enhancing activity of the very small, 33-residue P17 peptide tag for four previously-characterized scFvs expressed in *E. coli*. Importantly, the tag also increased stability of the *E. coli* expressed scFv. Furthermore, we characterized the features of the P17 tag which promote both solubility and stability, and explored the underlying mechanisms, eventually leading us to propose a type I intramolecular chaperone activity of the P17 tag. To our knowledge, the P17 tag is the first small peptide tag to increase both solubility and stability of scFv. We consider these as truly novel findings for prokaryotic scFv production which will benefit both basic research and applications of scFv.

2. There are some publications have studied the MBP, GST, Trx, et al tag for the fusion to improve the expression of scFv, also they get the good results. So why the author to select this P17 tag? The author compared P17 to MBP. Have you compare to other tag?
R2: We suppose "good results" refers to "good soluble expression" or "good solubility". Generally, high solubility of a protein indicates proper folding. Major parameters attributed to proper scFv folding are interactions between variable regions of heavy and light chains (VH and VL), and the formation of intra-domain disulfide bonds (Protein Science, 2017, 26, 1138-1149; Biochemistry, 1992, 31: 1270-1279). Indeed, some publications have studied fusions of MBP, Trx, and other tags to improve soluble expression of a few select scFvs in common *E. coli* strains such as BL21. The reducing cytoplasm in these strains prevents intra-domain disulfide bond formation, hence the occasionally observed partly soluble expression of certain scFvs likely relies on the strong intramolecular VH-VL interactions. However, many scFvs with suboptimal VH-VL interactions remain insoluble in common *E. coli* strains (Microbial Cell Factories, 2019, 18: 5) even when fused with bulky protein tags. For example, G12-scFv

was insoluble in *E. coli* BL21 star cells (Fig. 1b) and C-terminal MBP fusion failed to improve the solubility in SHuffle cells  (Fig. 3).

Although fusion of 43-kDa MBP or other bulky tags was reported to partly improve solubility of a few select scFvs, proteolytic removal of these bulky tags is often mandatory because otherwise they may interfere with scFv-antigen binding, impair tissue penetration and other pharmacokinetic properties of small-size scFv. In turn, removal of these bulky tags often destabilizes scFvs and induces non-functional aggregate formation.

Besides, due to the lack of inter- and intra-chain disulfide-bonds and other non-covalent interactions provided by the Fc domain, scFvs are less stable than mAbs with an increased tendency to unfold and aggregate under thermal stress. Therefore, intrinsically low solubility and stability remain major challenges for prokaryotic expression of scFvs.

Only 33-aa P17 peptide from the tail fiber protein of T7 phage was firstly reported by Dr. Jon A. Wolff's lab to target proteins and other cargos to hepatocytes and hepatocyte targeting activity of the P17 tag can be disabled by K26E mutation (Molecular Pharmaceutics, 2006, 3: 386-397). To improve hepatocyte uptake, we attached the P17 tag to the C terminus of G12-scFv binding the small surface protein of hepatitis B virus. When expressed in *E. coli* SHuffle cells, P17 tag increased the solubility of G12-scFv and three other scFvs by up to 11.6 fold. Importantly, P17 tag also increased thermo-stability of G12-scFv. Furthermore, P17 tag increased antigen-binding affinity and HBV neutralization activity of G12-scFv. **Since 4.1-kDa P17 tag is much smaller than other bulky protein tags to reduce metabolic burden of producer cells, harbors the unique feature of hepatocyte targeting,  and potently increases both solubility and stability of *E. coli*-produced scFv, in this study we aimed to identify the sequence and structure determinants within the P17 tag that are responsible for its solubility and stability enhancing impact on scFvs, and to clarify the underlying mechanisms.**

Following the reviewer's suggestion, GST tag was tested by fusing it to N terminus of G12-scFv since N-terminal but not C-terminal GST fusion was reported to promote soluble expression of passenger proteins (Malhotra, Meth. Enzymol., 2009, 463: 239-258). Results showed that GST tag reduced solubility of G12-scFv in *E.coli* SHuffle strain instead (Fig. 3).

3. E.coli BL21 (DE3) is a usually used host cell for expression, but the solubility is not good in BL21 this paper. SHuffle T7 is good for expression as in Fig.1. So if SHuffle T7 has the potential to express the scFv in large scale?

R3: Yes, we determined the soluble yields of P17-tagged G12-scFv and untagged version to be 8 mg and 3 mg per liter culture of *E. coli* SHuffle T7 cells (see Fig. S3b), respectively. As reported by us and others (Antiviral Research, 2019, 162: 118-129; Microbial Cell Factories, 2012, 11: 56; Biological & Pharmaceutical Bulletin, 2017, 40: 1767-1774; Nature Communications, 2015, 6: 8072), SHuffle cells have been used to express scFvs in large scale with yields of milligrams per liter *E. coli* culture.

4. In Fig.2. HA tag seems to have a negative effect than only P17, so only His tag can purify this fusion protein efficiently?

R4: Yes, the additional presence of an HA tag reduced solubility enhancement by the P17 tag (Fig. 1d and Fig. 2; see also reviewer #1, minor point 7). The HA tag was originally present in our constructs as an alternative means for immunodetection and interaction analysis of different scFvs. However, the N terminal His-tag performed equally well in such applications and also led to similar overall expression levels of different constructs, likely by providing for a similar translation initiation environment (ribosome-binding site/AUG/5´terminal codons). Moreover, the His-tag enabled efficient scFv purification using immobilized metal ion affinity chromatography, as shown in Fig. S3, and due to its small size it would not be expected to impair antigen binding by the scFv part. Hence for future applications of P17 tagged scFvs the HA tag is completely dispensable.

5. In Fig.4, only one P17 tag seems better. why two or three tags together is not good?

R5: Yes, one P17 tag increased scFv solubility reproducibly better than two or three copies (Fig. 4). Our initial rationale was that if solubility enhancement by the P17 tag works through recruiting chaperones (protein, or RNA - see reviewer #2, major point 4) then this effect might be even larger with more copies of P17 tags. However, experimentally we found a negative correlation. A plausible explanation could be sterical hindrance between the multiple tags but this would have to be further validated. Importantly for the current study only one copy of the P17 tag was able to efficiently increase the solubility of multiple scFvs in SHuffle cells.

6. In Fig.7, the authors mutated 4 amino acids to A, why only select this four?

R6: As outlined in our responses to Reviewers #1, minor point 9 and #2, major point 4 there was a possibility that solubility enhancement by P17 involves putative α-helix formation by residues 22-29 (the only region with a reasonable α-helix propensity, see new Suppl. Fig. 1); such a helix would feature two charged dipeptide motifs, $D_{23}E_{24}$, and $K_{26}R_{27}$. Moreover, our deletion analysis showed that variant P17-N18del, comprising the 15 C terminal residues including the putative α-helix sequence, was nearly as efficient as the full-length P17 tag in enhancing scFv solubility (Fig. 6 and 7). Hence the double-A replacements of $D_{23}E_{24}$, $K_{26}R_{27}$ and the combination of both within the P17-N18del context provided an opportunity to test for an influence of the clustered charged residues as part of a putative α-helix (see proposal by reviewer #2 of tRNA recruitment) yet also without such a structural context; the rationale here was the reported solubility enhancement for some recombinant proteins by polyanionic and polycationic tags (Microorganisms, 2018, 6: 47). Replacement of the charged residues, in particular of $D_{23}E_{24}$, and all four residues together did indeed substantially reduce solubility enhancement, by 42%, and 58%, respectively (Fig. 7d).

Together with the only minor negative impact of replacing the central A25 by a helix-breaking P residue mutation in wt P17 this supports our conclusion that charged and polar residues in P17, but not α-helix formation, are major contributors to the solubility enhancing effect. As notified by the reviewer, we added a few words “Because two anionic residues (D23, E24) and two cationic residues (K26, R27) are clustered in the central region of P17-N18del” in Line 293 of the revised version to clarify the reason why these four amino acids were mutated.

7. There are many important issues to be addressed for scFv, such as specificity, stability, affinity, solubility, et al. So the author should improve these issues.

R7: We agree that besides solubility (see point 1 above) many other issues for scFv production deserve further improvements but we respectfully disagree that our study would fall short of providing such data. As for solubility we had already shown in the initial submission that the P17 tag increased solubility of multiple (rather than just one) scFvs in *E. coli* SHuffle cells (Fig. 1-7), and documented by thermal shift assays the increased thermostability of *E. coli*-produced G12-scFv when tagged with the P17 tag (see Fig. 8). As for affinity, biolayer interferometry showed a 2.2-fold lower dissociation constant for the P17-tagged version of G12-scFv-HA (Fig. 9a), reflecting stronger binding affinity to hepatitis B surface antigen (HBsAg), likely by a larger fraction of properly folded molecules and/or their higher stability.

However, to independently confirm these data in a different assay format we now performed additional ELISA experiments (see also Reviewer #2, minor point 4). Microtiter wells were coated with 5 μg/ml of 12-scFv-HA with or without P17 tag (or anti-HBs from the KHB HBsAg kit as positive control and milk as negative control), and then reacted with pooled sera from HBsAg-positive chronic hepatitis B patients, or sera from healthy donors, or PBS. For the HBsAg-positive samples the $A_{450nm}$ values with G12-scFv-HA-P17 were significantly ($p<0.001$) nearly 2-fold higher than with untagged G12-scFv-HA (Fig. 9b), corroborating a stronger antigen-binding affinity. Conversely, the background $A_{450nm}$ values for the healthy donor sera (containing many non-HBV proteins) and PBS controls were significantly lower for the P17 tagged scFv (Fig. 9b), in line with increased specificity. The related description was added in Lines 344 to 356 of the current version.

REVIEWER COMMENTS

Reviewer #1 (Remarks to the Author):

I would like to begin by thanking the authors for taking the time to meticulously respond to every single comment by the reviewers. I sincerely think the current version is in much better ‘shape’ than the initial manuscript. That said, I regretfully would like to emphasize the importance of the major point I raised.

As the laboratory that has invented MBP fusion and studied it extensively for decades, we have extensive knowledge and experience with this solubility factor. It is accepted by the scientific community both for scientific and empirical reasons that the golden standard for using MBP as a solubility fusion is N-terminal and not C-terminal. It is therefore misleading to conclude that the P17 tag is better at solubilizing scFv than the MBP fusion, until the N-terminal fusion has been tested. I insisted on this experiment because should the N-terminal MBP fusion improve scFv by hundreds of fold (as has been observed in some cases) than the conclusions reached by this study would change drastically. Though all of the data currently presented would still stand, a reader would conclude that N-terminal MBP is the best option to improve scFv.

In fact, a brief literature search revealed the following:

“Evaluation of scFv protein recovery from E. coli by in vitro refolding and mild solubilization process” Animesh Sarker, Abhishek Singh Rathore & Rinkoo Devi Gupta

Microbial Cell Factories volume 18, 5 (2019)

https://microbialcellfactories.biomedcentral.com/articles/10.1186/s12934-019-1053-9

The authors also cited this publication, acknowledging… “Multiple fusion tags including MBP have been applied to improve the solubility of scFvs in E. coli27, 28, 29.”, but the authors have failed to state that the best improved (as expected) was MBP and was N-terminal fusion.

Further, MBP is known notoriously as ‘postponing the problem’ as it can solubilize a lot of proteins, but upon cleavage results in the precipitation of its cargo protein. As the fusion can sometime be a soluble IB. Thus cleavage of MBP and demonstration of the functionality of scFv by binding assays will also be required.

I would like to finish by saying that I am not in the habit of asking for extra experiments that do not challenge the scientific findings of manuscripts. However, in this one case I must make an exception. Without the industry golden standard of N-terminal fusion of MBP to scFv, the relative usefulness of P17 tag to the most common solubility factor cannot be evaluated.

Reviewer #2 (Remarks to the Author):

The revision is thorough, addressing to major issues and queries. The text is now greatly improved to increase the readability and minimize potential misunderstandings.

Reviewer #3 (Remarks to the Author):

The authors have carefully revised the manuscript according to my suggestions. So this revision could be accepted in the current form.

We thank all three reviewers for their positive comments on the revised version of our manuscript. Reviewer #2 and #3 had no more concerns but Reviewer #1 requested additional experimentation comparing scFv solubility enhancement by the P17 tag to that of N terminally, not only C terminally, fused maltose-binding-protein (MBP).

Unfortunately, due to anti-Corona measures including a lock-down in Shanghai since beginning of April 2022 we had no laboratory access for two months and access is still restricted. We tried our best but could only perform a limited number of new experiments; however, we feel that these new experiments, documented in the re-revised manuscript and discussed below, support our conclusion that the small P17 tag can be a highly valuable alternative also to N-terminally fused MBP, "the industry golden standard", as detailed below.

**Reviewer #1 (Remarks to the Author):**

I would like to begin by thanking the authors for taking the time to meticulously respond to every single comment by the reviewers. I sincerely think the current version is in much better 'shape' than the initial manuscript. That said, I regretfully would like to emphasize the importance of the major point I raised.

As the laboratory that has invented MBP fusion and studied it extensively for decades, we have extensive knowledge and experience with this solubility factor. It is accepted by the scientific community both for scientific and empirical reasons that the golden standard for using MBP as a solubility fusion is N-terminal and not C-terminal. It is therefore misleading to conclude that the P17 tag is better at solubilizing scFv than the MBP fusion, until the N-terminal fusion has been tested. I insisted on this experiment because should the N-terminal MBP fusion improve scFv by hundreds of fold (as has been observed in some cases) than the conclusions reached by this study would change drastically. Though all of the data currently presented would still stand, a reader would conclude that N-terminal MBP is the best option to improve scFv.

In fact, a brief literature search revealed the following:

"Evaluation of scFv protein recovery from E. coli by in vitro refolding and mild solubilization process" Animesh Sarker, Abhishek Singh Rathore & Rinkoo Devi Gupta Microbial Cell Factories volume 18, 5 (2019)
https://microbialcellfactories.biomedcentral.com/articles/10.1186/s12934-019-1053-9

The authors also cited this publication, acknowledging… "Multiple fusion tags including MBP have been applied to improve the solubility of scFvs in E. coli27, 28, 29.", but the authors have failed to state that the best improved (as expected) was MBP and was N-terminal fusion.

Further, MBP is known notoriously as 'postponing the problem' as it can solubilize a

lot of proteins, but upon cleavage results in the precipitation of its cargo protein. As the fusion can sometime be a soluble IB. Thus cleavage of MBP and demonstration of the functionality of scFv by binding assays will also be required.

I would like to finish by saying that I am not in the habit of asking for extra experiments that do not challenge the scientific findings of manuscripts. However, in this one case I must make an exception. Without the industry golden standard of N-terminal fusion of MBP to scFv, the relative usefulness of P17 tag to the most common solubility factor cannot be evaluated.

**Response:** We thank Reviewer #1 again for acknowledging the improvements of our revised manuscript, and given the widespread use of MBP as a solubility factor and the reviewer´s expertise, we appreciate the request to include N terminally fused MBP in our comparisons with the P17 tag. Hence we generated a new construct encoding an MBP-tag fused to the scFv N terminus, and determined the solubility of the encoded MBP-G12-scFv protein, its soluble yield, and compared its binding affinity to its cognate antigen, HBsAg in serum from chronic hepatitis B patients, with that of G12-scFv-HA-P17 and untagged G12-scFv-HA.

Indeed, N terminal but not C terminal MBP fusion increased the solubility of G12-scFv by 1.4-fold in SHuffle T7 cells (new Fig. 3c) as revealed by the solubility assay (new Figs. 3a and 3b). In comparison, the C terminal P17 tag increased solubility of G12-scFv by 1.7-fold, in line with a modestly higher soluble yield of G12-scFv-P17 than G12-scFv-HA and MBP-G12-scFv (Fig. S3b).

Previous reports have indicated that both N and C terminal MBP fusion can markedly improve solubility of some scFvs (Journal of Molecular Biology, 2001, 312: 79-93 and BMC Biotechnology, 2006, 6: 46) although, as stated by the reviewer, N terminally fused MBP may often be more effective. In our new data, the enhancement of G12-scFv solubility was indeed higher by N terminal than by C terminal MBP fusion but still slightly weaker than that by a C terminal P17 tag. This is not too surprising as solubility enhancement by MBP depends on the amino acid sequence of a passenger protein.

While solubility enhancement is one important feature, another is biological activity of the fusion protein. For instance, in a previous study an *E. coli* produced MBP-scFv fusion protein showed enhanced solubility compared to the untagged scFv but weaker specific antigen binding (BMC Biotechnology, 2006, 6: 46), presumably by steric interference by the bulky MBP tag. Our new data confirmed this phenomenon since, at identical molar ratios, binding to HBsAg of MBP-G12-scFv was significantly lower than that of untagged G12-scFv-HA and G12-scFv-HA-P17 as determined by ELISA (new Fig. S5). This could be due to steric interference by the bulky MBP tag but also to what the reviewer refers to as the "post-poning problem" of MBP (see above) which can keep incompletely and/or improperly folded passenger proteins in a soluble state,

e.g. as "soluble inclusion bodies"; removal of the MBP tag can then result in precipitation of the released passenger protein. We had already done such experiments using a G12-scFv-ETK-P17 construct carrying an enterokinase (ETK) recognition site between scFv and the C terminal P17 tag which demonstrated efficient and specific cleavage (Fig. S3 c).

We would have liked to follow the reviewer´s suggestion to perform analogous experiments with the new MBP-scFv construct but due to the strict anti-Corona measures in Shanghai we had to focus on few experiments. Importantly, our previous protease experiments with G12-scFv-ETK-P17 had already shown that after complete cleavage of the P17 tag essentially all of the released G12-scFv remained soluble, as indicated by the presence of unaltered protein amounts in the supernatant after 30 min centrifugation at 20,000 g. Hence while a direct comparison with removal of the N-terminal MBP tag would have been interesting, no significant further improvement regarding the yield of soluble tag-free G12-scFv could be expected. Hence we do not regard the lack of these experiments as critical for our conclusions.

Accordingly, we modified the manuscript by incorporating the results of the additional new experiments; the changes are marked in RED in the re-revised manuscript.

**Reviewer #2 (Remarks to the Author):**

**The revision is thorough, addressing to major issues and queries. The text is now greatly improved to increase the readability and minimize potential misunderstandings.**

**Response:** Thank you very much for the positive comments!

**Reviewer #3 (Remarks to the Author):**

**The authors have carefully revised the manuscript according to my suggestions. So this revision could be accepted in the current form.**

**Response:** Thank you very much for the positive comments!

REVIEWERS' COMMENTS

Reviewer #1 (Remarks to the Author):

Thank you for conducting the N-terminal MBP fusion experiment. I really think it was important to compare to your P17 tag. The manuscript looks good now.

Good luck
